# PROTACs: A novel strategy for cancer drug discovery and development

**DOI:** 10.1002/mco2.290

**Published:** 2023-05-29

**Authors:** Xin Han, Yi Sun

**Affiliations:** ^1^ Cancer Institute (Key Laboratory of Cancer Prevention and Intervention China National Ministry of Education) of the Second Affiliated Hospital and Institute of Translational Medicine Zhejiang University School of Medicine Hangzhou China; ^2^ Cancer Center of Zhejiang University Hangzhou China; ^3^ Zhejiang Provincial Clinical Research Center for CANCER Zhejiang Province China; ^4^ Key Laboratory of Molecular Biology in Medical Sciences Zhejiang Province China; ^5^ Research Center for Life Science and Human Health Binjiang Institute of Zhejiang University Hangzhou China

**Keywords:** E3 ligase ligands, PROTAC degraders, targeted therapy

## Abstract

Proteolysis targeting chimera (PROTAC) technology has become a powerful strategy in drug discovery, especially for undruggable targets/proteins. A typical PROTAC degrader consists of three components: a small molecule that binds to a target protein, an E3 ligase ligand (consisting of an E3 ligase and its small molecule recruiter), and a chemical linker that hooks first two components together. In the past 20 years, we have witnessed advancement of multiple PROTAC degraders into the clinical trials for anticancer therapies. However, one of the major challenges of PROTAC technology is that only very limited number of E3 ligase recruiters are currently available as E3 ligand for targeted protein degradation (TPD), although human genome encodes more than 600 E3 ligases. Thus, there is an urgent need to identify additional effective E3 ligase recruiters for TPD applications. In this review, we summarized the existing RING‐type E3 ubiquitin ligase and their small molecule recruiters that act as effective E3 ligands of PROTAC degraders and their application in anticancer drug discovery. We believe that this review could serve as a reference in future development of efficient E3 ligands of PROTAC technology for cancer drug discovery and development.

## Graphical abstract

We summarized the existing RING‐type E3 ubiquitin ligase and their small molecule recruiters to act as effective E3 ligands of PROTAC degraders. This review could serve as a reference for future development of efficient E3 ligands for PROTAC technology.

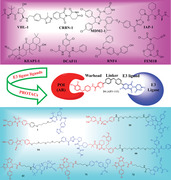

## 1 INTRODUCTION

Discovery of effective small molecule drugs for targeted therapy face several major challenges due to developed drug resistance, undruggable targets and poor selectivity among target family members.^1^, ^2^, ^3^, ^4^, ^5^, ^6^, ^7^, ^8^ Small‐molecule substitutes have emerged and gained clinical significance.^9^, ^10^, ^11^ The most representative alternative approach is the development of targeted protein degradation (TPD), with proteolysis targeting chimeras (PROTACs) as a typical example.^12^, ^13^, ^14^, ^15^, ^16^, ^17^, ^18^ TPD has entered its third decade with both opportunities and challenges.^19^, ^20^, ^21^, ^22^, ^23^, ^24^, ^25^, ^26^, ^27^, ^28^ TPD techniques rely on the ubiquitin–proteasome system (UPS), which couples ubiquitylation, catalyzed by E1 activating enzyme, E2 conjugate enzyme and E3 ligase, and proteasome for degradation of target substrates.^29^, ^30^, ^31^, ^32^, ^33^, ^34^, ^35^, ^36^, ^37^, ^38^, ^39^, ^40^, ^41^

**FIGURE 1:**
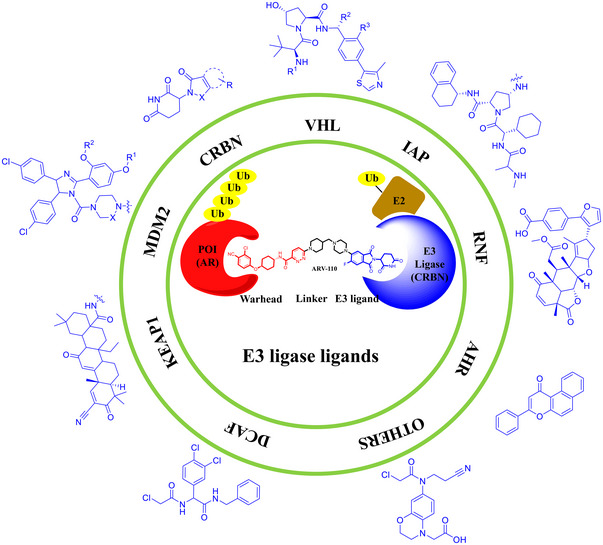
A summary of current ligands for few E3 ubiquitin ligases used in PROTACs (PDB ID: 7B5S). Structures or general structural formulas of these E3 ligands and a working model of PROTAC with ARV‐110 as an example.

The concept of PROTAC was first proposed in 2001, and the PROTAC technology has developed rapidly in the past 20 years.^5^, ^8^, ^19^, ^42^, ^43^, ^44^, ^45^ At the present, more than 10 PROTAC degraders have entered clinical Phase I‐II trials with ARV‐471 and ARV‐110 from Arvinas, Inc as the most advanced ones for the treatment of recurrent breast cancer and prostate cancer, respectively.^46^, ^47^, ^48^, ^49^ However, PROTAC still faces many challenges.^50^, ^51^, ^52^, ^53^, ^54^, ^55^, ^56^, ^57^, ^58^, ^59^, ^60^, ^61^, ^62^, ^63^, ^64^, ^65^ A typical PROTAC degrader consists of three components: the target‐binding small molecule ligand (protein of interest, POI), the E3 ligase ligand and the appropriate linker that connects the two components (Figure 1).^50^, ^66^, ^67^, ^68^, ^69^, ^70^, ^71^, ^72^, ^73^ E3 ligases and the corresponding ligands are important as the driving force of protein degradation.^74^, ^75^, ^76^, ^77^, ^78^, ^79^, ^80^, ^81^, ^82^, ^83^, ^84^, ^85^, ^86^, ^87^, ^88^, ^89^, ^90^, ^91^ Good drug‐like small‐molecule ligands for a E3 ligase system are still limited.^92^, ^93^, ^94^, ^95^, ^96^, ^97^ Although more than 600 E3 ligases are encoded by human genome, only less than 10 E3 ligases were developed as ligands for the development of PROTAC degraders, including von Hippel‐Lindau (VHL), cereblon (CRBN), mouse double minute 2 (MDM2), cellular IAP1 (cIAP1), Kelch‐like ECH‐associated protein‐1 (KEAP1), DDB1‐cullin 4‐associated factor (DCAF), RING finger protein (RNF), aryl hydrocarbon receptor (AHR), and others (Figure 1).^66^, ^74^, ^98^, ^99^, ^100^, ^101^, ^102^, ^103^, ^104^ Therefore, it is urgent to develop new and efficient E3 ligands to expand the role of PROTAC technology in drug discovery and development.^105^, ^106^, ^107^, ^108^, ^109^, ^110^, ^111^, ^112^ In this review, we systematically summarize all reported E3 ligases and their corresponding small molecule ligands for the successful development of PROTAC degraders.

## 2 VHL LIGANDS AND THEIR UTILIZATIONS IN PROTACS FOR CANCER DRUG DISCOVERY

### 2.1 Common VHL ligands

**FIGURE 2:**
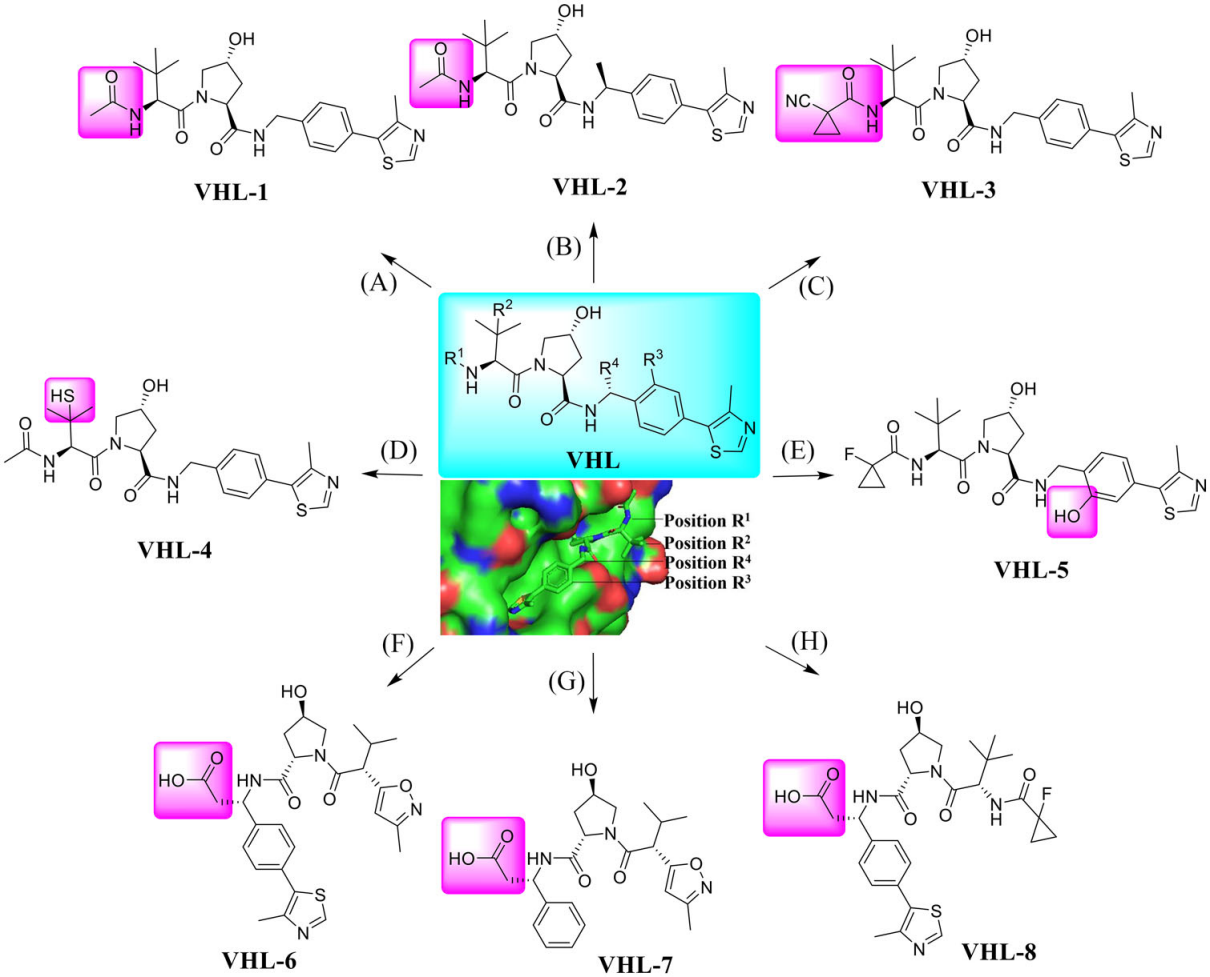
A variety of small molecules that serve as the ligands for VHL E3 ligase. (A–C) The VHL ligands retain the R^1^ tethering position based upon a cocrystal structure of a VHL‐1 ligand in complex with pVHL:EloB:EloC (PDB ID: 4W9H). (D) The VHL ligand retains the R^2^ tethering position. (E) The VHL ligand retains the R^3^ tethering position. (F‐H) The VHL ligands retain the R^4^ tethering position.

VHL protein, together with elongin B and C, Cullin 2, and RBX‐1, is part of a multiprotein complex with E3 ubiquitin ligase activity.^113^, ^114^, ^115^, ^116^, ^117^, ^118^ In the complex, VHL is responsible for binding to specific substrates, most notably hypoxia‐inducible factor (HIF‐1 α), for its ubiquitination and proteasome degradation.^119^, ^120^ In 2012, Crews group disclosed the first small‐molecule ligands for VHL E3 ligase based on skeleton of hydroxyproline.^121^ Since then, a series of novel and highly effective VHL E3 ligase ligands have been discovered and reported, typified by compounds VHL‐1–VHL‐8 (Figure 2) with improved lipophilicity.^119^, ^120^, ^122^ The studies on the eutectic structure of the VHL ligand with the protein helps to locate the solvent‐exposed region, leading to revelation of four possible linking sites without negatively affecting the interaction between the protein and the corresponding ligand (Figure 2; PDB ID: 4W9H).^123^, ^124^, ^125^, ^126^, ^127^, ^128^, ^129^, ^130^, ^131^, ^132^, ^133^, ^134^ These sites are (a) terminal amino; (b) sulfhydryl; (c) benzyl; and (d) phenolic hydroxyl group on the benzene ring.^113^, ^135^, ^136^, ^137^, ^138^, ^139^, ^140^, ^141^ At present, VHL E3 ligand has been widely and successfully applied in the design and synthesis of PROTAC as one of the most commonly used E3 ligands (Table 1).^13^, ^17^, ^18^, ^142^, ^143^, ^144^, ^145^, ^146^, ^147^, ^148^, ^149^, ^150^, ^151^, ^152^, ^153^, ^154^

**TABLE 1:** Chemical structures and biological activities of representative PROTAC degraders.

No.	Name	Structure	DC_50_ (μM)	POI	E3 ligand
1	ARCC‐4	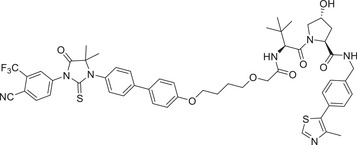	0.005 (VCaP)	AR	VHL‐1/VHL‐3
2	PROTAC‐1	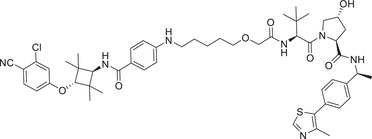	NA^a^	AR	VHL‐2
3	ARD‐61	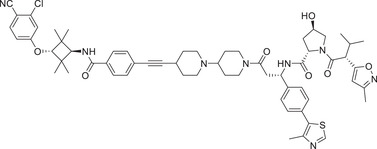	0.001 (VCaP)	AR	VHL‐6
4	ARD‐69	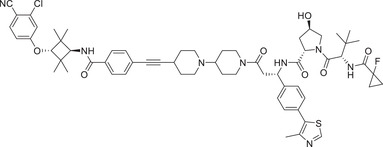	0.00074 (VCaP)	AR	VHL‐8
5	ARD‐266	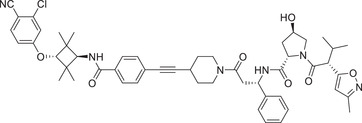	0.001 (VCaP)	AR	VHL‐7
6	SJF‐0628	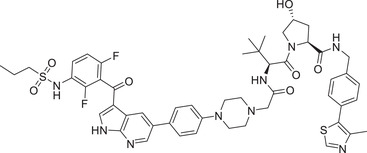	0.0068 (SK‐MEL‐28)	BRAF	VHL‐1/VHL‐3
7	MS‐39	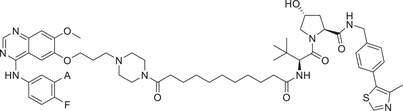	0.0033 (H3255)	EGFR	VHL‐1/VHL‐3
8	14o	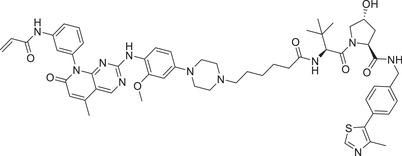	0.0059 (H1975)	EGFR	VHL‐1/VHL‐3
9	PROTAC‐2	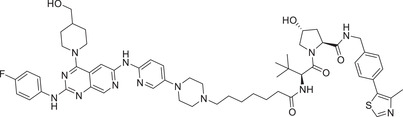	0.0348 (HCC‐827)	EGFR	VHL‐1/VHL‐3
10	LC‐2	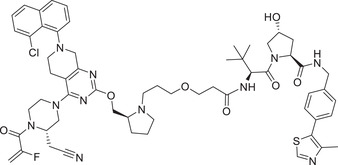	0.59 (NCI‐H2030)	KRAS	VHL‐1/VHL‐3
11	YF‐135	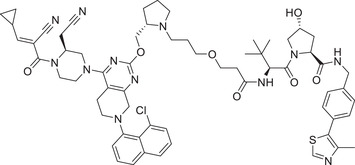	3.61 (NCI‐H358)	KRAS	VHL‐1/VHL‐3
12	GMB‐805	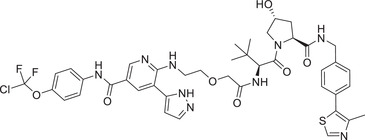	0.03 (K562)	BCR–ABL	VHL‐1/VHL‐3
13	SIAIS178	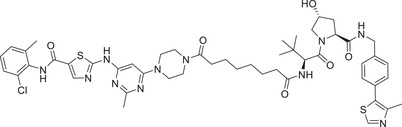	0.0085 (K562)	BCR–ABL	VHL‐1/VHL‐3
14	JNJ‐1013	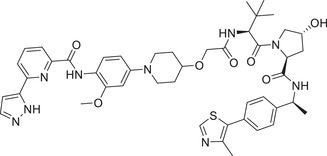	0.003 (HBL‐1)	IRAK1	VHL‐2
15	PROTAC‐3	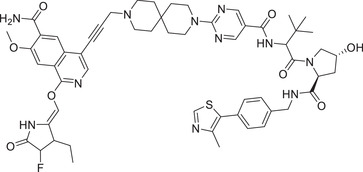	0.15 (PBMC)	IRAK4	VHL‐1/VHL‐3
16	ERD‐308	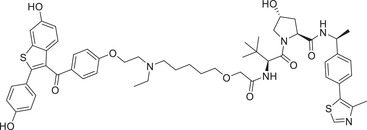	0.00017 (MCF‐7)	ER	VHL‐2
17	AM‐A3	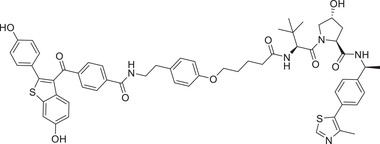	0.0011	ER	VHL‐2
18	PROTAC‐4	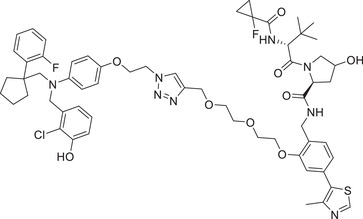	0.037	ER	VHL‐5
19	SHP2‐D26	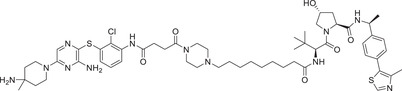	0.0026 (MV4; 11)	SHP2	VHL‐2
20	AT‐1	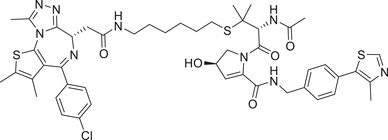	0.1 (Hela)	BRD4	VHL‐4
21	AGB1	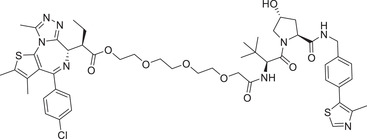	0.015	BRD4	VHL‐1/VHL‐3
22	3j	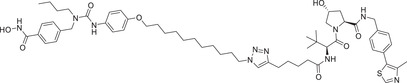	0.0071 (MM.1S)	HDAC	VHL‐1/VHL‐3
23	XH‐07‐189	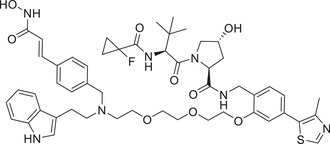	NA	HDAC	VHL‐5
24^b^	ARV‐110^b^	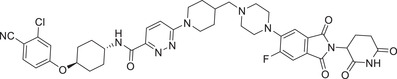	0.0016 (VCaP)	AR	CRBN‐1/CRBN‐2
25	PROTAC‐5	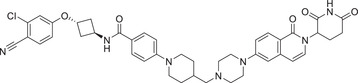	NA	AR	CRBN‐12
26	ARD‐2128	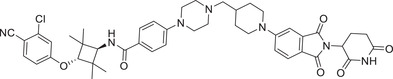	0.00028 (VCaP)	AR	CRBN‐1/CRBN‐2
27	ARD‐2585	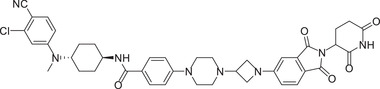	0.00004 (VCaP)	AR	CRBN‐1/CRBN‐2
28	MS910	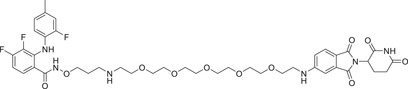	0.094/MEK1; 0.038/MEK2 (SK‐MEL‐28)	MEK	CRBN‐1/CRBN‐2
29	CPS2	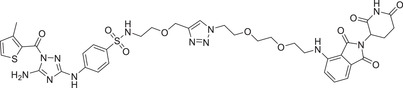	0.002 (MV4; 11)	CDK2	CRBN‐1/CRBN‐2
30	TMX‐2172	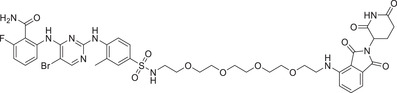	NA	CDK2/5	CRBN‐1/CRBN‐2
31	PROTAC‐6	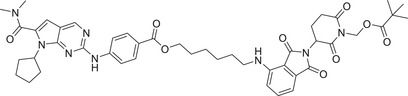	NA	CDK2/4/6	CRBN‐5
32	B03	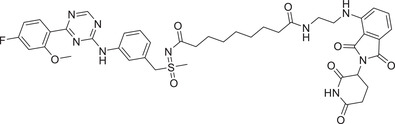	0.0076 (MV4;11)	CDK9	CRBN‐1/CRBN‐2
33	SIAIS001	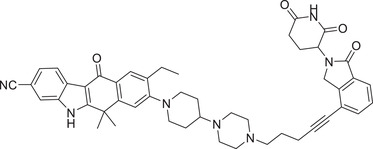	0.0039 (SR)	ALK	CRBN‐3
34	PROTAC‐7	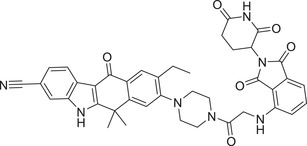	0.027 (H3122)	ALK	CRBN‐1/CRBN‐2
35	MD‐224	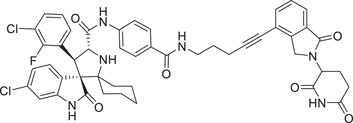	0.001 (VCaP)	MDM2	CRBN‐3
36	WB214	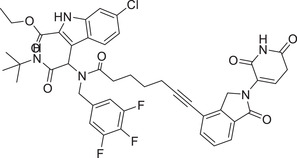	0.0041 (RS4;11)	MDM2	CRBN‐3
37	INY‐03‐041	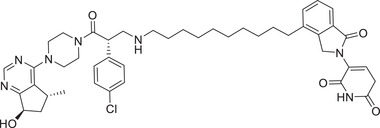	NA	AKT	CRBN‐3
38	MS170	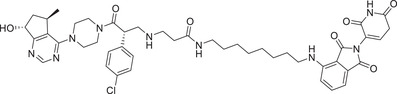	0.032 (BT474)	AKT	CRBN‐1/CRBN‐2
39	SD‐36	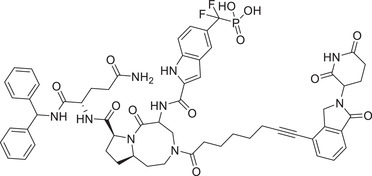	0.06 (Molm‐16)	STAT3	CRBN‐3
40	AK‐2292	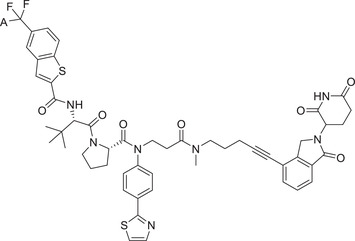	0.1 (SKNO1)	STAT5	CRBN‐3
41^c^	ARV‐471^c^	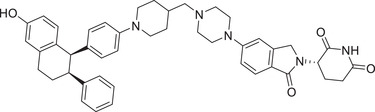	0.002 (MCF‐7)	ER	CRBN‐4
42	PROTAC‐8	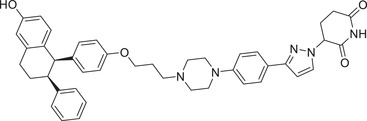	NA	ER	CRBN‐11
43	SIAIS125	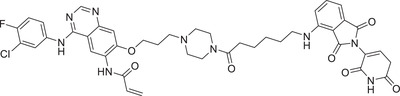	0.1 (PC9)	EGFR	CRBN‐1/CRBN‐2
44	PROTAC‐9	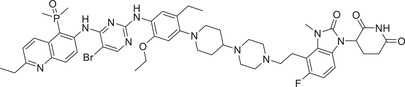	NA	EGFR	CRBN‐6
45	QCA570	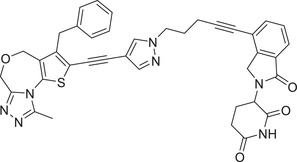	<0.00001 (RS4;11)	BET	CRBN‐3
46	PROTAC‐10	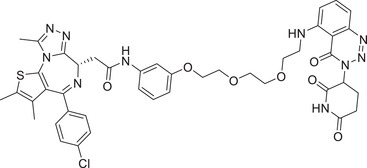	0.00032 (22Rv1)	BET	CRBN‐7
47	PROTAC‐11	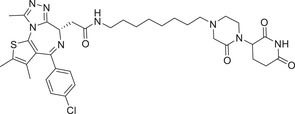	NA	BET	CRBN‐10
48	SJ10542	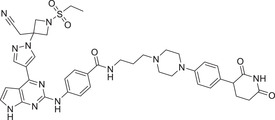	0.014 (JAK2) 0.011 (JAK3) (PDX cells SJBALL020589)	JAK2/3	CRBN‐9
49	DGY‐08‐097	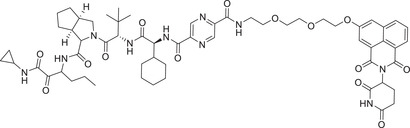	0.05 (HCV‐NS3)	HCV	CRBN‐8
50	PROTAC‐12	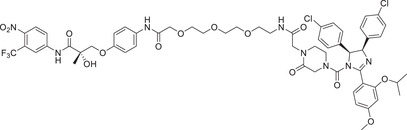	10 (Hela)	AR	MDM2‐1
51	A1874	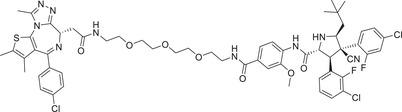	0.023 (HCT116)	BRD4	MDM2‐2
52	PROTAC‐13	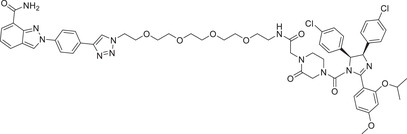	5 (MDA‐MB‐231)	PARP1	MDM2‐3
53	PROTAC‐14	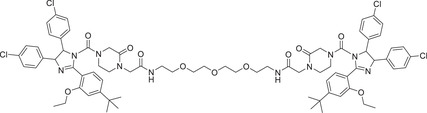	NA	MDM2	MDM2‐1/MDM‐4
54	PROTAC‐15	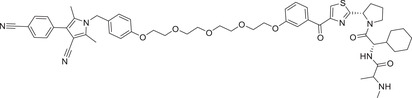	2 (22Rv1)	AR	IAP‐1
55	PROTAC‐16	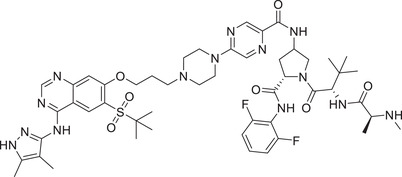	7.9 (THP‐1)	RIPK2	IAP‐2
56	PROTAC‐17	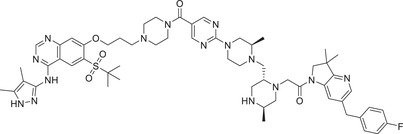	1.6 (THP‐1)	RIPK2	IAP‐3
57	PROTAC‐18	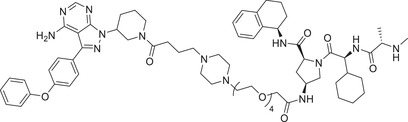	0.2 (THP‐1)	BTK	IAP‐4
58	BCPyr	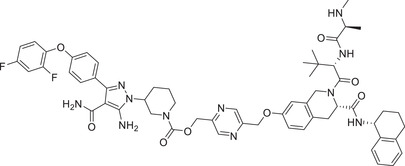	0.8 (THP‐1)	BTK	IAP‐6
59	PROTAC‐19	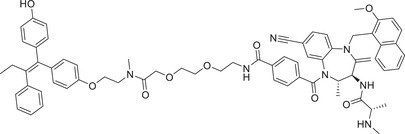	0.68 (MCF‐7)	ER	IAP‐5
60	SNIPER‐2	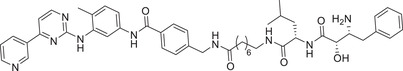	30 (K562)	BCR–ABL	IAP‐7
61	PROTAC‐20	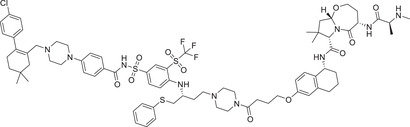	0.1 (MyLa 1929)	BCL‐X_L_	IAP‐8
62	CDDO‐JQ1	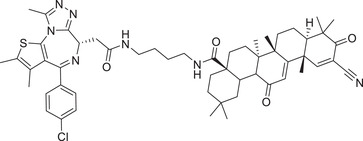	0.05 (231MFP)	BRD4	KEAP1‐1
63	MS‐83	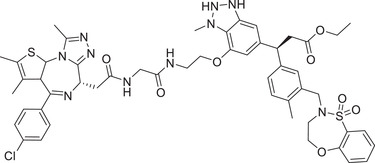	0.25 (MDA‐MB‐231)	BRD2/3/4	KEAP1‐3
64	PROTAC‐21	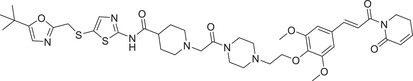	0.009 (MOLT4)	CDK9	KEAP1‐2
65	KB02‐SLF	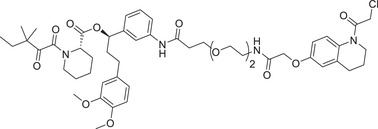	0.5 (HEK293T)	FKBP12	DCAF16
66	21‐SLF	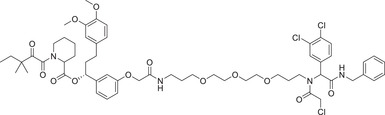	NA	FKBP12	DCAF11
67	YT117R	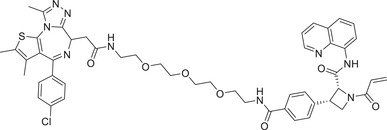	NA	BRD4	DCAF1
68	DP‐1	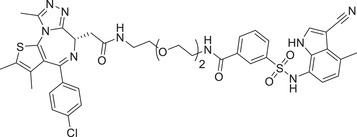	10.8 (SU‐DHL‐4)	BRD2/3/4	DCAF15
69	XH‐2	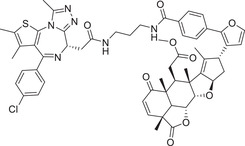	0.5 (231MFP)	BRD4	RNF114
70	ML 2‐14	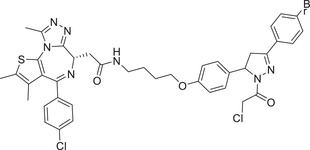	0.014 (231MFP)	BRD4	RNF114 (EN219)
71	CCW 28‐3	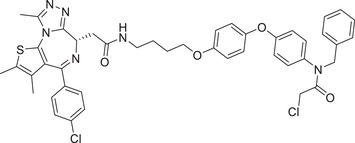	0.1 (231MFP)	BRD4	RNF4
72	NJH‐1‐106	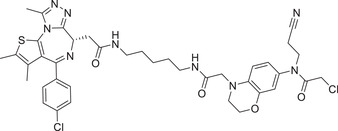	0.25 (HEK293T)	BRD4	FEM1B
73	KL‐7	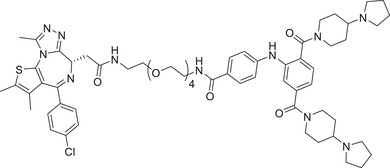	5 (HCT116)	BRD2	L3MBTL3
74	KL‐4	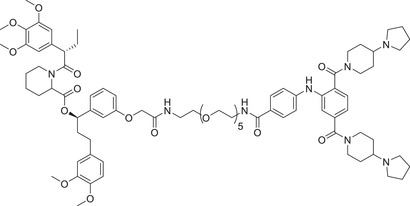	1 (HEK293_HFNES)	FKBP12^F36V^	L3MBTL3
75	BT‐1	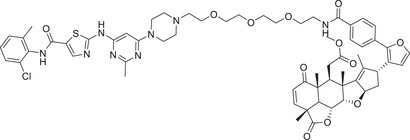	1 (K562)	BCR–ABL	RNF114
76	β‐NF‐ATRA	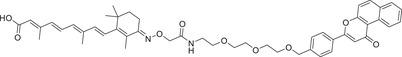	NA	CRABPs	β‐NF

^a^ Not Applicable.

^b^ Phase II.

NCT03888612.

^c^ Phase III, NCT05654623.

Abbreviations: AKT, protein kinase B; ALK, anaplastic lymphoma kinase; AR, androgen receptor; BCL‐XL, recombinant human B‐cell leukemia/lymphoma XL; BCR–ABL, breakpoint cluster region‐Abelson; BET, the bromo and extral terminal domain family; BRAF, v‐raf murine sarcoma viral oncogene homolog B1; BRD4, bromodomain‐containing protein 4; BTK, Bruton's tyrosine kinase; CDK, cyclin‐dependent kinase; CRABPs, cellular retinoic acid binding proteins; CRBN, cereblon; DC_50_, half‐maximal degradation concentrations; DCAF, DDB1‐cullin 4‐associated factor; EGFR, epidermal growth factor receptor gene; ER, estrogen receptor; FKBP12, FK506 binding protein 12 kDa; HCV, hepatitis C virus; HDAC, histone deacetylase; IAP,  inhibitor of apoptosis protein; IRAK, interleukin‐1 receptor‐activated kinase; JAK, Janus kinase; KEAP1, the Kelch‐like ECH‐associated protein‐1; KRAS, Kirsten rat sarcoma viral oncogene; MDM2, murine double minute 2; MEK, mitogen activated protein kinase; PARP1, poly(ADP‐ribose) polymerase; POI, protein of interest; RIPK2, receptor Interaction serine/threonine kinase 2; RNF, RING finger protein.; SHP2, Src homology region 2‐containing protein tyrosine phosphatase 2; STAT, signal transducer and activator of transcription; VHL, Von Hippel‐Lindau.

### 2.2 The utilizations in PROTACs

A variety of PROTAC degraders have been designed and discovered based on the four potential binding sites of VHL ligands.

#### 2.2.1 AR

As an important member of the nuclear hormone receptor superfamily, androgen receptor (AR) plays a crucial role in the maintenance and development of male prostatic secondary sex characteristics.^155^ The dysfunction of AR is a major cause for the development of human prostate cancer. At the present, metastatic castration‐resistant prostate cancer (mCRPC) remains incurable and fatal. AR antagonists are effective for the treatment of mCRPC, significantly improves the survival of prostate cancer patients with well tolerance.^156^, ^157^ A plenty of AR antagonists have been developed for the treatment of advanced prostate cancer, exemplified by enzalutamide. In prostate cancer patients, resistance to enzalutamide usually appears within 18 months.^158^, ^159^, ^160^ The AR signaling pathway remained functional in the majority of patients with enzalutamide resistance. Targeting AR protein degradation is a very promising therapeutic strategy for the treatment of mCRPC, particularly enzalutamide‐resistant mCRPC with potential being more effective than AR antagonists.

In 2018, a PROTAC AR degrader named ARCC‐4 (Table 1) was reported by Crews group, using VHL‐1/VHL‐3 (Figure 2).^161^ ARCC‐4 showed good in vitro potency including low‐nanomolar DC_50_ (half‐maximal degradation concentrations) value, moderate inhibition on proliferation of prostate cancer cells with better antiproliferative effects on enzalutamide‐resistant AR mutant cells. In 2019, Arvinas’ patent disclosed a series of potent PROTACs targeting AR and Kargbo, showing that the chiral methyl group in VHL ligand (Figure 1; VHL‐2) improved the degradation capacity, as exemplified by PROTAC‐1 (Table 1).^162^ Recently, the Wang group discovered a series of new VHL ligands (Figure 1; VHL‐6–8) in the process of designing AR degraders ARD‐61, ARD‐69, and ARD‐266 (Table 1).^163^, ^164^, ^165^, ^166^ The (*S*)‐methyl group in VHL‐2 is exposed to the solvent zone, which can be used as a possible tethering point in the design of PROTAC AR degraders, based on the cocrystal structure of VHL‐1 and VHL protein (Figure 2). Compounds ARD‐61 and ARD‐69 effectively induced the degradation of AR with DC_50_ values as low as 0.74–1 nM in VCaP cells. These two compounds also showed excellent prostate cancer cell growth inhibition in vitro and tumor inhibition in vivo.

#### 2.2.2 BRAF

The RAF kinase family mainly regulates cell proliferation, growth, differentiation, survival, and other physiological activities via the RAS–RAF–MEK–ERK signaling pathway.^167^ Mutational activation of BRAF, in a form of BRAF^V600E^, occurs in many human cancers, which is associated with the occurrence and development of human cancers.^168^ Drug resistance severely limits the clinical effectiveness of BRAF*^V600E^* inhibitors. In addition, BRAF*^V600E^* small molecule inhibitors mainly act by binding with the catalytic bag of RAF with incomplete inhibition, because they failed to inhibit the BRAF dimer, another key mechanism of RAF activation.^169^, ^170^ New drug discovery techniques, such as PROTAC, are urgently needed to overcome the limitations of existing RAF inhibitors and provide the basis for new targeted alternative therapy strategies for the treatment of BRAF*^V600E^*‐causing cancer.

In 2021, Crews group disclosed a series of VHL‐based degraders by coupling BRAF inhibitor vemurafenib to VHL ligands (Figure 1; VHL‐1/VHL‐3), as exemplified by compound SJF‐0628 via a rigid piperazine linker (Table 1).^171^ BRAF degrader SJF‐0628 could effectively induce degradation of BRAF*^V600E^*, but not wild type BRAF protein in multiple cell lines. The DC_50_ of SJF‐0628 for BRAF*^V600E^* was impressive 6.8 nM in SK‐MEL‐28 cell line, SJF‐0628 also has good inhibitory effect on other tumor cells.^171^

#### 2.2.3 EGFR

Epidermal growth factor receptor (EGFR) is a glycoprotein of tyrosine kinase‐type receptors, which consists of three regions: the intracellular kinase region, the transmembrane region and the extracellular ligand‐binding region.^172^ EGFR is involved in a variety of physiological activities, overexpression, and abnormal activation in a variety of solid tumors, and is closely related to angiogenesis, cell proliferation, tumorigenesis, migration, invasion, and tumor metastasis.^173^, ^174^ Three generations of small molecule EGFR inhibitors have been approved by the United States Food and Drug Administration (US FDA) to treat non‐small cell lung cancer (NSCLC) and other human cancers, severe resistance in clinical patients due to persistent isomerism mutations (EGFR*^C797S^*) remains an insurmountable problem for these inhibitors. PROTAC is a revolutionary technology to overcome the resistance of EGFR inhibitors for effective cancer therapy.

In 2019, Jin group developed a novel class of EGFR degraders based VHL E3 ligase ligands (Figure 1; VHL‐1/VHL‐3), as exemplified by compound MS‐39 (Table 1).^175^ Compound MS‐39 could effectively induce the degradation of EGFR mutants with good selectivity but not EGFR*^WT^* with DC_50_ value of 3.3 nM in H3255 (EGFR*^L858R^*) cells. This compound also inhibited the proliferation of other lung cancer cells. In addition, MS‐39 is a potential candidate compound for in vivo pharmacodynamic study due to its good PK properties. In 2020, Ding group reported a class of new PROTACs based on a selective EGFR*^L858R/T790M^* inhibitor (XTF‐262) that can efficiently target EGFR*^L858R/T790M^* degradation.^176^ They compared the abilities of four E3 ligases (VHL, CRBN, cIAP1, and MDM2) in the design of novel EGFR degraders, and found that degradation of EGFR*^L858R/T790M^* was effectively induced by compound 14o (Table 1) with VHL ligand (Figure 1; VHL‐1/VHL‐3) and DC_50_ value of 5.9 nM in H1915 cell line. In 2020, Zhang group reported a series of EGFR degraders based on VHL E3 ligands (Figure 1; VHL‐1/VHL‐3).^177^, ^178^ The most potent degrader PROTAC‐2 (Table 1) induced degradation of EGFR with a DC_50_ value of 34.8 nM in HCC‐827 (EGFR*^e19d^*) cell line.

#### 2.2.4 KRAS

Rat sarcoma virus oncogene homologues (RAS) and KRAS are among the most common RAS genes associated with human cancer.^179^ The mutational activation of KRAS is one of most frequently events occurring in human cancers and causally related to tumorigenesis, thus KRAS is an effective antitumor drug target.^180^ The discovery of KRAS drugs has been challenged in past 30 years because it has a flat structure without approachable pocket for small molecules to bind, thus being an undruggable target.^181^, ^182^, ^183^, ^184^ Few small molecules targeting KRAS‐G12C, known as the switch II Pocket (G12C) via covalent binding, was recently approved in May 2021 by Amgen (AMG510‐sotorasib) and Mirati (MRTX849‐adagrasib) for clinical trials.^185^ However, it has not been possible to rapidly develop inhibitors of other KRAS onco‐alleles, such as G12D, which is dominant in pancreatic cancer and new drug development strategies still need to be explored.

In 2020, Crews group reported endogenous KRAS*^G12C^* degradation by connecting the covalent KRAS inhibitor MRTX849 to VHL ligands (Figure 1; VHL‐1/VHL‐3).^186^ After screening, compound LC‐2 (Table 1) was identified as the most potent KRAS*^G12C^* degrader with a *D*_max_ value of 80% and a DC_50_ value of 0.59 μM in inducing degradation of endogenous KRAS*^G12C^* in NCI‐H2030 cell line. At the same time, Lu group reported a new series of KRAS*^G12C^* degraders based on the structure of LC‐2. In their study, the promising compound YF‐135 (Table 1) could induce the degradation of KRAS*^G12C^* protein with a moderate DC_50_ value of 3.61 μM in the H358 cells.^187^ Compound YF‐135 is the first reversible covalent PROTAC degrader that can induce KRAS*^G12C^* degradation through recruitment of VHL‐mediated proteasome.

#### 2.2.5 BCR–ABL

BCR/ABL fusion gene is an antiapoptotic gene with high tyrosine kinase activity, which leads to overproliferation of cells and disorder of cell regulation.^188^ Constitutively active BCR–ABL activates the downstream proliferative signaling pathways to cause chronic myelogenous leukemia (CML).^189^ Currently, three generations of BCR–ABL inhibitors have been approved to treat CML, mainly including Imatinib (Gleevec), Nilotinib, and Ponatinib. However, drug resistance develops after initial success and various side effects also limit its clinical application.^190^, ^191^, ^192^ Therefore, the discovery of BCR–ABL degraders seems to overcome these problems.

In 2016, Crews group disclosed the first Dasatinib‐based BCR–ABL PROTAC degrader, while this compound achieved only micromolar (>60% at 1 μM) degradation of BCR–ABL, and unable to overcome generic resistant mutants, especially for the T315I mutant.^193^ Subsequently, the same group successively designed and synthesized a new VHL‐based (Figure 1; VHL‐1/VHL‐3) BCR–ABL PROTAC degrader GMB‐805 (Table 1),^194^ which had more than ten‐times increase in inducing BCL–ABL degradation with improved pharmacokinetic properties and in vivo activity. In 2019, Jiang group combined the same BCR–ABL inhibitor Dasatinib and VHL E3 ligand (Figure 1; VHL‐1/VHL‐3) through extensive optimization of the linker to discover a new class of BCR–ABL degraders.^195^ The compound SIAIS178 (Table 1) was identified as the most promising BCR–ABL degrader to induce an effective degradation of wild‐type BCR–ABL in K562 cells as well as several clinically relevant drug‐resistant mutations with a DC_50_ value of 8.5 nM.

#### 2.2.6 IRAK

Interleukin‐1 receptor‐activated kinase (IRAK) plays a critical role in signal transduction of innate immune responses.^196^ The IRAK kinase family consists of four members, IRAK1, IRAK2, IRAK3, and IRAK4, which are potential targets for therapeutic interventions in autoinflammatory diseases.^197^, ^198^, ^199^ IRAK1 is a kind of serine‐threonine protein kinase that involves in many downstream signal transduction of TLRs and IL‐1R, and is an effective target for drug development for the treatment of chronic inflammatory diseases. Despite some progress in the development of IRAK1 inhibitors, IRAK1 remains a very challenging target due to the lack of a domain primarily responsible for its scaffold function.^200^ Furthermore, the role of IRAK4 in tumor growth and progression and the mode of action for IRAK4 inhibitors are still unclear.^201^

In 2021, the Dai group reported that an IRAK1 degrader JNJ‐1013 can effectively degrade cellular IRAK1 protein with a DC_50_ value of 3 nM in HBL‐​1 cell line (Table 1 and Figure 2; VHL‐2).^202^ In addition, JNJ‐1013 could effectively inhibit the downstream signaling pathway of IRAK1 and showed strong antiproliferation effect in activated B‐cell‐like (ABC) DLBCL cell line mutated with MyD88.^202^ This study shows that IRAK1 degrader have the potential to treat cancers dependent on the function of IRAK1 stents compared to IRAK1 inhibitors. In 2019, Anderson group developing a series of PROTACs by binding the IRAK4 inhibitor PF‐06650833 to different E3 ligases.^203^ The most effective degrader of IRAK4 PROTAC‐3 (Table 1) in PBMC cells was induced by VHL‐based E3 ligands (Figure 1; VHL‐1/VHL‐3) with a DC_50_ value of 151 nM.

#### 2.2.7 ER

Estrogen receptors (ER) are located in the nucleus and consist of two types of classical nuclear receptors, ERα and ERβ, which mediate the effects of estrogen.^204^, ^205^ Studies have shown that ER is closely related to the occurrence and development of breast cancer, in which the overexpression of ER α (ER‐positive) accounts for 70% of breast cancer,^206^ thus being a well‐established target for the treatment of breast cancer.^207^ Currently approved endocrine treatments for breast cancer include aromatase inhibitors (AIs), like letrozole, selective ER modulators (SERMs), like tamoxifen, and selective ER degraders (SERDs), like fulvestrant.^208^ However, drug resistance caused by long‐term clinical use limited their therapeutic efficacy. Furthermore, the oral bioavailability of existing breast cancer drugs, such as selective ER degrader fulvestrant, can only be administered by intramuscular injection.^209^, ^210^ The emergence of PROTACs provides a new approach for the discovery and development of ERα targeted drugs.

In 2019, Wang group developed a new class of highly potent ER degraders, as exemplified by compound ERD‐308 (Table 1 and Figure 2; VHL‐2).^211^ ERD‐308 effectively reduced the degradation of ER to achieve DC_50_ values of 0.17 and 0.43 nM in MCF‐7 and T47D ER+ breast cancer cells, respectively. More importantly, ERD‐308 induced more complete ER degradation than fulvestrant, the only SERD approved by the US FDA, and achieved better cell growth inhibition than fulvestrant in MCF‐7 cell line. In 2020, Tang group designed and developed a series of ER degraders based on PROTACs strategy using the same ER antagonist and VHL ligand in compound ERD‐308.^212^ The promising compound AM‐A3 (Table 1) showed a good degradation activity on ER with a DC_50_ value of 1.1 nM and a *D*_max_ value of 98% in MCF‐7 cell line. In addition, AM‐A3 can also effectively induce potent antiproliferation with an IC_50_ value of 13.2 nM in MCF‐7 cells. In 2021, X‐Chem Inc. disclosed a new class of ER PROTAC degraders based on ER antagonist and VHL E3 ligand (Figure 2; VHL‐5) with a new linking position on the phenolic hydroxyl group.^213^ With the DNA‐encoded chemical library screening, they found a promising compound PROTAC‐4 (Table 1) which could induce the degradation of ERα with a DC_50_ value of 37 nM and inhibit the growth of MCF‐7 cells efficiently.

#### 2.2.8 SHP2

Protein tyrosine phosphatase Src homology region 2‐containing protein tyrosine phosphatase 2 (SHP2) mutations in the Src homologous domain of the protein tyrosine phosphatase family exist in most tumor cells and are closely associated with cancer development and poor prognosis of cancer patients^214^, ^215^ by activating several proliferative signaling pathways for cancer occurrence.^216^ Thus, SHP2 was proposed as an attractive target for cancer treatment.

In 2020, Wang group reported a novel class of SHP2 degraders by conjugating with the SHP2 inhibitor SHP099 and the VHL ligand VHL‐2 (Figure 2).^217^ The most potent compound SHP2‐D26 (Table 1) effectively induced the degradation of SHP2 protein with a DC_50_ value of 2.6 nM in MV4;11 cells and with *D*_max_ to reduce SHP2 protein levels in cancer cells by more than 95%. In addition, compound SHP2‐D26 showed 30‐fold more potent than the SHP2 inhibitor SHP099 in MV4;11 cells.

#### 2.2.9 BRD

The bromo and extral terminal domain family (BET) proteins, including BRD2, BRD3, BRD4, and BRDT, are a class of bromodomain‐containing proteins that have been extensively studied.^218^ Over the years, a large number of inhibitors targeting BET proteins have been developed, which have good anticancer efficacy, and some compounds have successfully entered clinical studies.^219^, ^220^ However, for highly homologous BET proteins, it is difficult to achieve highly selective targeting of BET subtypes. PROTAC technology can improve the selectivity of target protein degradation through the binding of triprimary complex and the recognition of polyubiquitination. Thus, it is a new strategy for the discovery and development of selective BET targeting drugs.

In 2017, Ciulli group reported a novel BRD4 degrader AT‐1 (Table 1) based on VHL ligand by linking on the sulfydryl of VHL‐4 (Figure 2).^221^ Recently, they also developed a novel class of BRD4 degraders based on VHL E3 ligands (Figure 2; VHL‐1/VHL‐3), as exemplified by compound AGB1 (Table 1). The degrader AGB1 could completely induce the degradation of BromoTagged target proteins with a low DC_50_ value of 15 nM and is highly selective to native wild type BET protein.^222^

#### 2.2.10 HDAC

Histone deacetylases (HDACs) removes acetyl‐group mainly from histones, thus acting as “epigenetic erases” to play a crucial role in modification of chromosome structure and regulation of gene expression.^223^ Frequent alterations of HDACs in human cancers make them attractive cancer targets for the discovery and development of small molecule inhibitors.^224^, ^225^ Indeed, HDAC inhibitors were found to induce cell cycle arrest, cell death, and differentiation, to blocked angiogenesis as well as to modulate immune response, and few inhibitors have been approved for the treatment of some T‐cell lymphoma and multiple myeloma.^226^

Currently, the major challenge of achieving complex selectivity with HDAC inhibitors is considered to be a major source of off‐target toxicity and adverse reactions associated with HDAC inhibitors.^227^, ^228^ Using PROTAC to improve selectivity may be an effective strategy to solve this problem.^229^, ^230^

In 2020, Tang group reported the first class of selective HDAC6 degraders employing VHL E3 ubiquitin ligase.^231^ The representative compound 3j (Table 1 and Figure 2; VHL‐1/VHL‐3) could effectively induce HDAC6 degradation with high selectivity, not have activity on any known neo‐substrates. The DC_50_ and *D*_max_ values of the most potent compound 3j are 7.1 nM and 90%, respectively, in human MM.1S cells. VHL‐5 (Figure 2) was also used in the discovery of HDAC degrader XH‐07‐189 through linking the phenolic hydroxyl by Fischer group in 2021 (Table 1).^232^

Although VHL E3 ligand was among the first in the development of PROTAC degraders, the difficulty in reaching the oral availability limited its clinical usage.^233^, ^234^

## 3 CRBN LIGANDS AND THEIR UTILIZATIONS IN PROTACS

### 3.1 Common CRBN ligands

**FIGURE 3:**
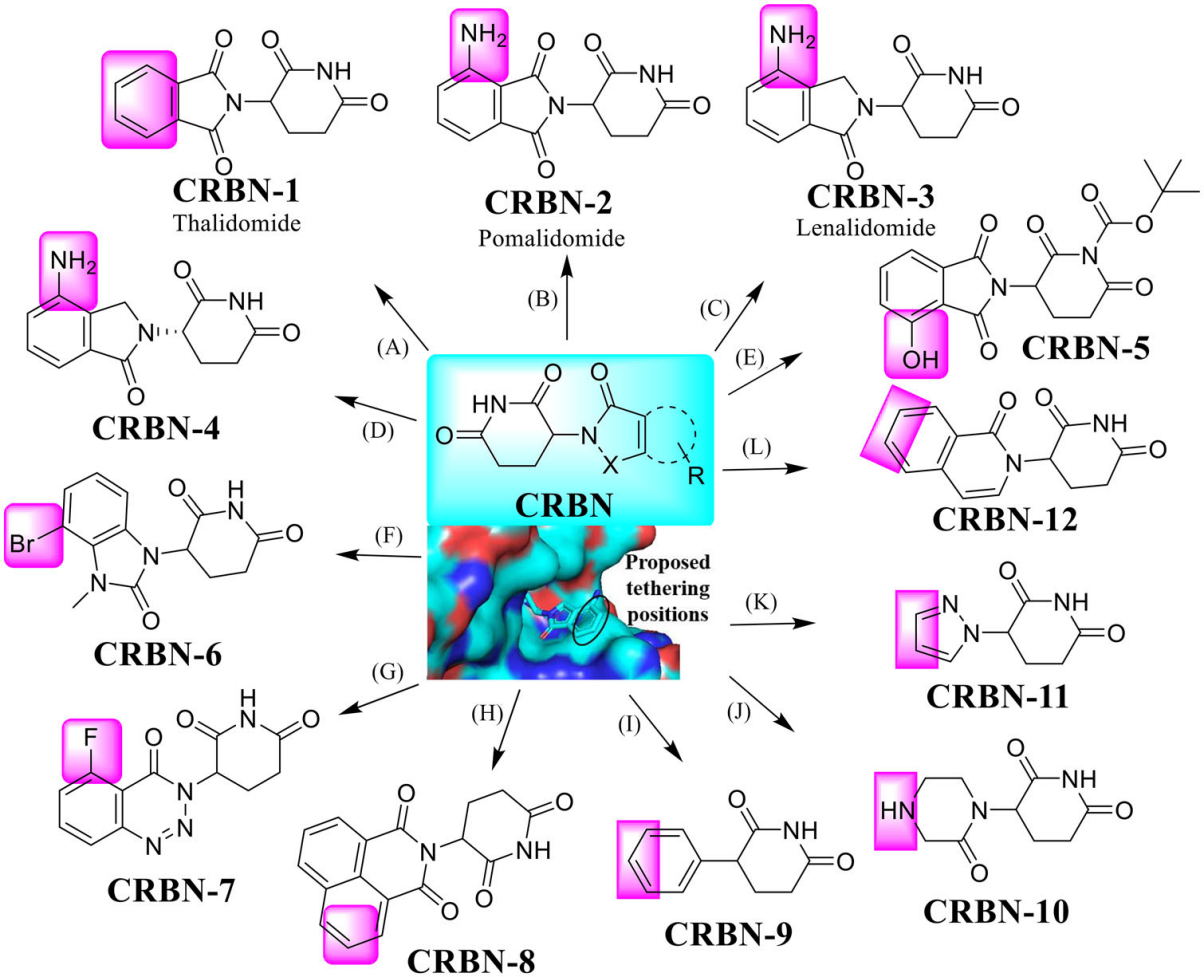
A variety of small molecules that serve as the ligands for CRBN E3 ligase. (A and B) Various small molecules acting as the ligands for CRBN E3 ligase and proposed tethering position in the CRBN ligand based upon a cocrystal structure of lenalidomide in complex with DDB1–CRBN–CUL4 E3 ubiquitin ligase (PDB ID: 4CI2). (C and D) The lenalidomide and its *S*‐isomer. (E) CRBN E3 ligand for prodrug strategy. (F) Imidazole‐CRBN E3 ligand. (G) Triazin‐CRBN E3 ligand. (H) Isoquinoline‐CRBN E3 ligand. (I) Phenyl glutarimide (PG)‐CRBN E3 ligand. (J) Oxopiperazin glutarimide‐CRBN E3 ligand. (K) Pyrazol glutarimide‐CRBN E3 ligand. (L) Oxoisoquinolin glutarimide‐CRBN E3 ligand.

CRBN is a protein composed of 442 amino acids that is substrate‐recognizing subunit, complexed with scaffold protein Cullin‐4 and adaptor protein: damaged DNA binding protein 1 (DDB1) to form the Cullin‐4‐RING ligase (CRL4).^235^, ^236^, ^237^, ^238^, ^239^, ^240^, ^241^ CRBN, a substrate receptor of CRL4, has been widely studied for its regulation of a variety of biological processes.^242^, ^243^, ^244^, ^245^, ^246^ The discovery of CRBN E3 ligase ligands can be traced back to the developed drug thalidomide (Figure 3) as sedative used to treat insomnia in the 1950s.^247^, ^248^, ^249^, ^250^, ^251^, ^252^, ^253^ However, it was quickly discontinued in clinic due to its teratogenic effects.^254^, ^255^ Although it has since been found to treat a number of other diseases, its molecular mechanism remains unclear.^256^, ^257^ It was not until 2010 that thalidomide was found to directly bind to CRBN, acting as an immunomodulatory drug (IMiD), for its mechanism of action.^4^, ^5^, ^50^, ^258^, ^259^, ^260^, ^261^, ^262^, ^263^, ^264^, ^265^ In the subsequent decade, the research based on thalidomide and its analogues (e.g., pomalidomide and lenalidomide) (Figure 3) has grown rapidly, mainly in the area of PROTAC and molecule glue.^67^, ^266^, ^267^, ^268^, ^269^, ^270^, ^271^, ^272^, ^273^, ^274^, ^275^, ^276^, ^277^, ^278^, ^279^, ^280^ The eutectic structure of lenalidomide and CRBN revealed that the exposure of lenalidomide benzene ring to the solvent region is a potential good binding site (Figure 3; PDB ID: 4CI2).^281^, ^282^, ^283^

### 3.2 The utilizations in PROTACs

A variety of PROTAC degraders have been discovered and developed based on the four potential binding sites of CRBN E3 ligands.^284^, ^285^

#### 3.2.1 AR

Thalidomide, one of the most classic CRBN E3 ligase ligands, has been successfully applied in the design and discovery of a variety of highly efficient PROTAC degraders, including the first clinical AR degrader ARV‐110 (Table 1 and Figure 3; CRBN‐1/CRBN‐2), the first two published oral AR degraders ARD‐2128 and ARD‐2585 (Table 1 and Figure 3; CRBN‐1/CRBN‐2).^47^, ^48^, ^49^, ^286^, ^287^ Compound ARV‐110 is an AR degrader drug discovered by Crews group and Arvinas, Inc. to overcome the developed drug resistance of prostate cancer. After further optimized dose escalation exposure with different AR antagonists, ARV‐110 completely degraded AR protein levels in all tested cell lines (LNCaP, VCaP, MCF7, etc.) with DC_50_ values <1 nM in vitro. In addition, in vivo trials as well as in prostate cancer resistant to enzalutamide showed good efficacy. ARV‐110 is currently in the clinical phase II in patients with mCRPC. In 2018, Arvinas, Inc. disclosed a patent to show a new type of AR degrader PROTAC‐5 (Table 1) using oxoisoquinolin glutarimide ligand CRBN‐12 (Figure 3) as the CRBN recruiter.^242^ In 2021, Wang group developed two class of potent AR degraders ARD‐2128 and ARD‐2585 with good oral bioavailability (Table 1 and Figure 3; CRBN‐1/CRBN‐2). The oral bioavailability of compounds ARD‐2128 and ARD‐2585 in mice reached 67 and 51%, respectively, which effectively reduced the level of AR protein and significantly inhibit tumor growth in mice without any observable toxicity. Furthermore, ARD‐2585 showed an excellent degradation ability in MDA‐PCa‐2b (*L701H* and *T877A*) cell lines, which laid a solid foundation for overcoming drug resistance.

#### 3.2.2 MEK

MEK1 and MEK2 (mitogen activated protein kinase) are two family members of MAP kinase, which are important signaling molecules in the Ras–RAF–MEK–ERK pathway.^288^ MEK1 and MEK2 activate downstream ERK to promote growth and survival of cancer cells, thus playing an important role in tumor development.^289^, ^290^

The Jin group has been engaged in the discovery of a variety of MEK degraders based on VHL and CRBN E3 ligands. Specifically, in 2020 the group reported several novel and potent VHL recruited MEK1/2 degraders, and the first CRBN recruited MEK1/2 degrader MS910 (Table 1). These compounds effectively and selectively degrade MEK1/2, thus inhibiting downstream signaling pathways and growth of cancer cells.^291^

#### 3.2.3 CDK

Currently, 20 proteins have been classified as members of the CDK family, a large part of which are widely recognized as key regulatory factors of cell cycle, transcription, metabolism and/or cell differentiation, and are closely related to the occurrence and development of tumors, with CDK2/4/6 as the best examples of cancer targets.^292^, ^293^ The inactivation of cyclin‐dependent kinase 2 (CDK2) effectively overcomes the blocking of cell differentiation in acute myeloid leukemia (AML) and is therefore a promising and potential approach for the treatment of AML.^294^

In 2021, the Rao group disclosed a highly potent CDK2 depressant CPS2 (Table 1) via the conjugated CRBN ligands (Figure 3; CRBN‐1/CRBN‐2) and the nonselective CDK2 inhibitor JNJ‐7706621.^295^ The compound CPS2 effectively induced rapid and efficient degradation of CDK2 with a DC_50_ value of 2 nM in MV4;11 cell line. In 2020, the Gray group developed a selective CDK2/5 degraders over the other CDKs, as exemplified by compound TMX‐2172 (Table 1 and Figure 3; CRBN‐1/CRBN‐2).^296^ Compound TMX‐2172 selectively induced the degradation of CDK2/5 in a time and dose‐dependent manner. Biologically, they demonstrated that degradation of CDK2 protein has antiproliferative activity in ovarian cancer cells (OVCAR8). In 2021, the Yang group developed a novel PROTAC CDK2/4/6 degrader PROTAC‐6 (Table 1 and Figure 3; CRBN‐5), based on the structure of CDK inhibitor Ribociclib's derivatives. Compound PROTAC‐6 was designed through prodrug strategy by introducing a leaving group.^297^ In 2021, the Bian group developed a series of selective CDK9 degraders by conjugating a selective CDK9 ligand BAY‐1143572 and CRBN ligand pomalidomide (Figure 3; CRBN‐1/CRBN‐2), as exemplified by compound B03 (Table 1). Compound B03 could also effectively induce the degradation of CDK9 with a DC_50_ value of 7.6 nM in MV4;11 cells.

#### 3.2.4 ALK

As a tyrosine kinase in the insulin receptor kinase subfamily, anaplastic lymphoma kinase (ALK) is a potent and promising oncogenic factor in lung cancer.^298^ Therefore, targeting ALK fusion proteins is an emerging and effective target for the treatment of cancer, especially NSCLC.^299^ However, the durability of clinical efficacy of ALK tyrosine kinase inhibitors in NSCLC is often limited by drug resistance within 1−2 years, partly due to acquired ALK resistance mutations.^300^, ^301^ Therefore, new effective treatment strategies are needed to overcome the defects of clinical resistance to ALK inhibitors.

In 2020, the Jiang group developed a series of ALK degraders based on ALK inhibitor Alectinib and lenalidomide (Figure 3; CRBN‐3).^302^ The promising compound SIAIS001 (Table 1) showed good ALK degradation activity with a DC_50_ value of 3.9 nM and a *D*_max_ of 70.3% in SR cell line. In 2021, the Xu group developed, based on the same ALK inhibitor Alectinib, a class of ALK degraders employing CRBN ligase.^303^ The promising compound PROTAC‐7 (Table 1 and Figure 3; CRBN‐1/CRBN‐2) could effectively induce the degradation of ALK with a DC_50_ value of 27 nM in H3122 NSCLC cell line.

#### 3.2.5 MDM2

MDM2 is an onco‐protein that acts as an E3 ubiquitin ligase to promote ubiquitylation and degradation of p53, a classical tumor suppressor, which induces growth arrest or apoptosis of cancer cells to suppress tumor formation.^304^, ^305^ Therefore, targeting MDM2 protein by chemical means would reactivate p53 as a promising strategy for the discovery and development of antitumor drugs.^306^, ^307^ Over the years, a number of small molecule inhibitors that disrupt MDM2‐p53 interaction have been reported and some were developed in clinic,^308^ but none of them has yet been approved by the US FDA for anticancer therapy. The cumulative toxicity of MDM2, as a result of p53 activation to transactivate MDM2 expression, is a major problem affecting the development of these MDM2‐p53 binding inhibitors.^309^ Thus, the discovery and development of PROTAC for targeted degradation of MDM2 is a promising strategy to overcome this side‐effect of small molecule inhibitors.

In 2019, the Wang group reported a PROTAC MDM2 degrader.^310^ The most active compound MD‐224 (Table 1 and Figure 3; CRBN‐3) effectively induced rapid degradation of MDM2 at the concentrations <1 nM in human leukemia cell lines. The compound showed good anticancer activity in both in vitro and in vivo. Specifically, compound MD224 achieved complete and lasting tumor regression in vivo with a well‐tolerated dose schedule in RS4;11 xenograft tumor models.^310^ In 2021, the Tang group disclosed a series of MDM2 degraders based on MDM2 inhibitors and lenalidomide (Figure 3; CRBN‐3).^311^ After extensive optimization, the most potent WB214 (Table 1) was shown to have an impressive MDM2‐degradation activity with DC_50_ value of 4.1 nM against leukemia cells.

#### 3.2.6 AKT

As a downstream component of the phosphoinositol 3‐kinase (PI3K) signaling cascade, serine/threonine kinase AKT positively regulates several key processes in cell proliferation, survival, and metabolism.^312^ AKT is overactivated by acquired functional mutation, amplification of upstream oncogenes (receptor tyrosine kinases and PI3K) or inactivation of tumor suppressor genes (PTEN, INPP4B, and PHLPP), which contributes to the malignant phenotypes associated with tumorigenesis.^313^ Therefore, targeting AKT is an attractive therapeutic strategy for cancer treatment.

In 2020, the Toker group developed a class of pan‐AKT degraders consisting of a recruiter of the ATP competitive AKT ligand GDC‐0068 coupled with CRBN ligand lenalidomide, CRBN‐3 (Figure 3).^314^ Compared with GDC‐0068, the most promising compound INY‐03‐041 effectively induced degradation of all three AKT isoforms and showed enhanced antiproliferative effects. Using the same GDC‐0068, the Jin group also developed a potent AKT degrader MS170 (Table 1), employing CRBN by pomalidomide ligands (Figure 3; CRBN‐1/CRBN‐2).^315^ The degrader MS170 showed AKT‐degradation activities with a DC_50_ value of 32 nM in BT474 breast cancer cells. Compound MS170 also showed good growth inhibition activity against different cancer cell lines, including PC‐3 prostate cancer cells, BT474 and MDA‐MB‐468 breast cancer cells.^314^

#### 3.2.7 STAT

Signal transducer and activator of transcription (STAT) is a unique family of proteins that can bind to DNA.^316^ The STAT family consists of STAT1, STAT2, STAT3, STAT4, STAT5, and STAT6.^317^, ^318^ STAT proteins, particularly STAT3 and 5 transduce signals from cytokines and growth factors through their receptors to the nucleus to regulate expression of a variety of genes, leading to cell proliferation, apoptosis inhibition, and chemo‐resistance^319^, ^320^ (PMID: 36596870). As promising classical targets for the treatment of human cancers, several small molecule inhibitors targeting STAT3 and STAT5 have entered clinical trials, but none of them have been in clinical use.^321^, ^322^, ^323^ PROTAC degraders targeting STAT3/5 has been recently developed.

In 2019, The Wang group first developed a novel STAT3 degrader SD‐36 (Table 1) by employing ligands for CRBN/cullin 4A E3 ligase (Figure 3; CRBN‐3) and STAT3 inhibitors.^324^, ^325^, ^326^ SD‐36 highly selectively induces rapid degradation of STAT3 at low nanomolar concentration in cancer cells. Significantly, complete and lasting tumor regression was achieved by compound SD‐36 in a Molm‐16 xenograft tumor model with a well‐tolerated dose schedule. Very recently, the same group reported a new class of highly effective STAT5 degraders, typically by compound AK‐2292 (Table 1 and Figure 3; CRBN‐3).^327^, ^328^ The most active compound AK‐2292 effectively induced degradation of STAT5A, STAT5B, and phosphorylated STAT5 proteins in AML cells in a concentration‐ and time‐dependent manner, and exhibited excellent degradation selectivity for STAT5 over all other STAT members. The in vivo tumor model study also showed that AK‐2292 effectively reduced STAT5 protein levels with strong antitumor activity under a tolerated dose schedule.^327^, ^328^

#### 3.2.8 ER

The compound ARV‐471 (Table 1), a potent oral ER degrader, was discovered by using the *S*‐isomer of lenalidomide CRBN‐4 (Figure 3).^46^, ^49^ In 2019, the ER‐targeted PROTAC ARV‐471 developed by Crews group and Arvinas, Inc. was approved by the US FDA as an investigational new drug for the treatment of locally advanced or metastatic ER‐positive/HER2‐negative breast cancer.^46^ At present, ARV‐471 has successfully passed the phase II clinical study and started the Phase III clinical study at the beginning of 2023, which is the furthest clinical progress of a degrader in the field of PROTAC technology. The same group also developed a new series of potent ER degrader PROTAC‐8 (Table 1) utilizing a pyrazol glutarimide ligand CRBN‐11 (Figure 3) as the CRBN recruiter described in their patent application.^242^

#### 3.2.9 EGFR

In 2021, Jiang group reported a novel class of highly selective and functional PROTAC degraders targeting EGFR, as exemplified by compound SIAIS125 (Table 1) based on Canertinib and CRBN E3 ligand (Figure 3; CRBN‐1/CRBN‐2).^329^ Compound SIAIS125 effectively induced the degradation of EGFR protein with a DC_50_ value of 100 nM in PC9 cell line. The Lei group disclosed a series of novel bifunctional compounds as EGFR degraders using CRBN‐6 (Figure 3) in 2022, as exemplified by compound PROTAC‐9 (Table 1).^330^

#### 3.2.10 BET

In 2018, the Wang group reported a new BET inhibitor in the class of [1,4]oxazepines, and then synthesized a new class of highly efficient small molecule BET degraders. Through systematic modification and activity screening, the optimal compound QCA570 (Table 1 and Figure 3; CRBN‐3) can effectively induce the degradation of BET protein and inhibit the growth of human acute leukemia cell line at low picmolal concentration. The in vivo study showed that QCA570 achieved complete and sustained tumor regression in a mouse leukemia xenograft model and was well tolerated. In 2019, the Hwang group disclosed a series of bifunctional compounds by conjugation of BET inhibitor JQ‐1 with a novel CRBN modulator (Figure 3; CRBN‐7), as exemplified by compound PROTAC‐10 (Table 1).^331^ Compound PROTAC‐10 could effectively induce the degradation of BET proteins with a good DC_50_ value of 0.32 nM in 22Rv1 cell line. In 2017, the C4 Therapeutics disclosed a patent to show various derivatives of glutarimide as novel CRBN ligands and their applications in discovering BET degrader, as exemplified by compounds oxopiperazin glutarimide‐CRBN‐10 (Figure 3) as CRBN recruiter and PROTAC‐11 as BET degrader (Table 1).^242^

#### 3.2.11 JAK2/3

Common Janus kinases (JAKs), JAK2 and JAK3, are involved in a variety of cell signal transduction related to T‐ and B‐cell‐mediated diseases and are closely associated with the pathogenesis of common lymphogenic diseases and leukemia.^332^, ^333^ Therefore, the development of targeted inhibitors or degraders by intervening in JAK2/3 is a valuable research strategy for reducing the risk of these diseases. In 2022, the Rankovic group developed a novel and potent PROTAC degrader targeting JAK2/3 kinases utilizing a phenyl glutarimide (PG) inhibitor as the CRBN recruiter.^243^ The most potent compound SJ10542 (Table 1 and Figure 3; CRBN‐9) could effectively induce the degradation of JAK2/3 with DC_50_ values of 14 nM and 11 nM in PDX cells. Compound SJ10542 can also inhibit the PDX cells (JAK2‐fusion ALL) with IC_50_ value of 24 nM.

#### 3.2.12 HCV

PROTAC techniques are also increasingly being used to degrade viral proteins for potential therapeutic virus‐related indications, like Hemagglutinin 1 Neuraminidase 1 virus (HIN1) and hepatitis C virus (HCV). Recently, the Yang group discovered a novel class of small molecule antivirals that induce proteasomal degradation of HCV proteins.^334^ The most promising degrader DGY‐08‐097 (Table 1 and Figure 3; CRBN‐8) could effectively induce the degradation of HCV proteins and contributes to the inhibition of HCV replication in cells.

These reports laid a solid foundation for future discovery of CRBN‐based degraders to treat a variety of human cancers. So far, CRBN‐based E3 ligands confer the best oral availability in all known PROTAC degraders, thus having high potential for clinical application.^46^, ^47^, ^286^, ^287^

## 4 MDM2 LIGANDS AND THEIR UTILIZATIONS IN PROTACS

### 4.1 Common MDM2 ligands

**FIGURE 4:**
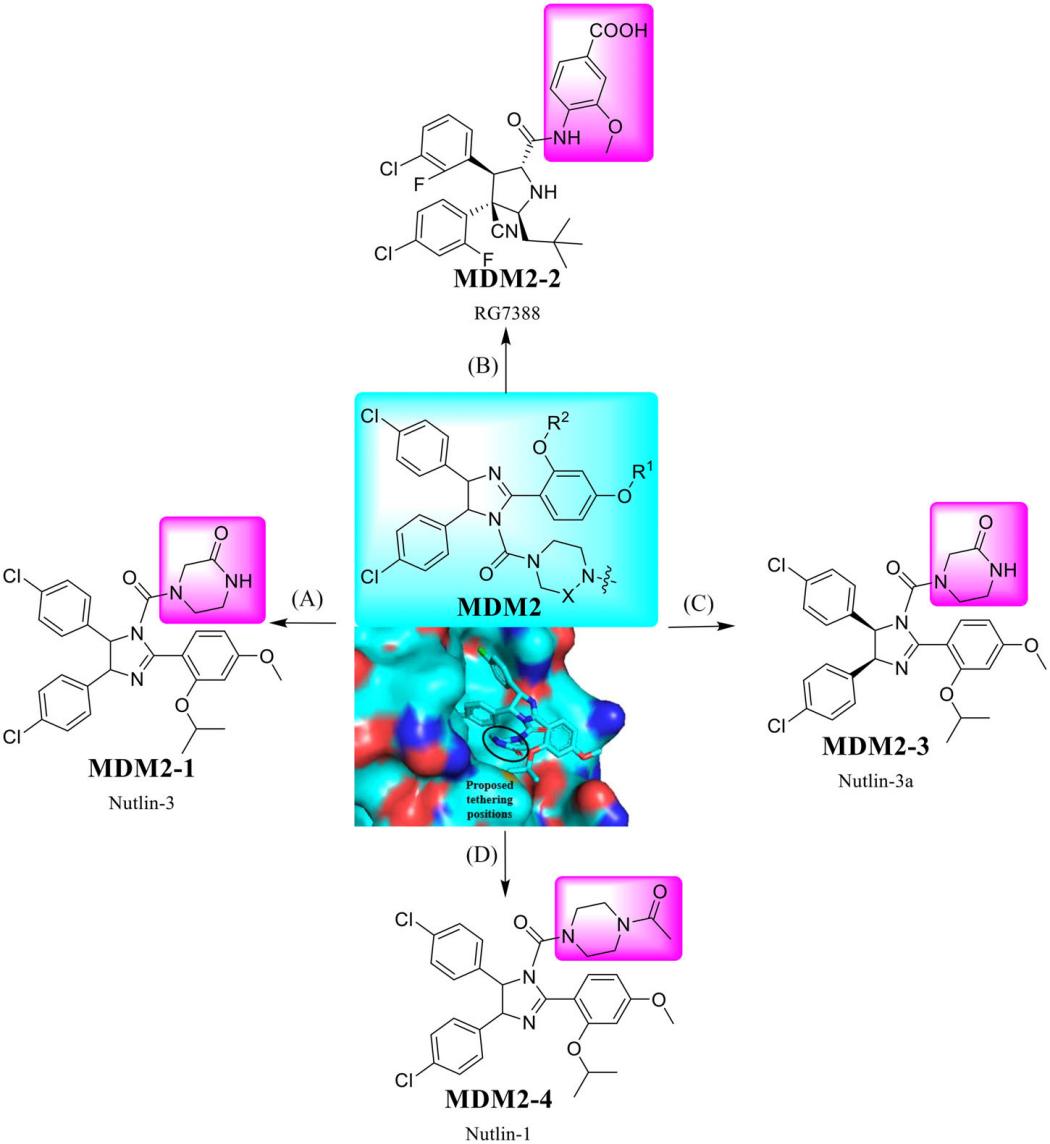
A variety of small molecules that serve as the ligands for MDM2 E3 ligase. (A‐D) Various small molecules (MDM2‐1–4) acting as MDM2 E3 ligase ligands and proposed tethering position in MDM2 ligand, based upon a co‐crystal structure of the MDM2 inhibitor nutlin‐3 and MDM2 protein (PDB ID: 4J3E).

MDM2 is an E3 ubiquitin ligase that promotes tumor development by binding to the tumor suppressor p53 for degradation.^335^, ^336^, ^337^, ^338^, ^339^, ^340^, ^341^, ^342^ Over the past 20 years, a number of MDM2 inhibitors were used in the discovery of PROTAC degraders, like RG7388 and Nutlin‐3 series (Figure 4).^19^, ^343^, ^344^, ^345^, ^346^ From the cocrystal structure of the MDM2 inhibitor nutlin‐3 with MDM2 (Figure 4; PDB ID: 4J3E),^347^ the pink moiety is exposed to the solvent and is a potentially ideal linking position in the design of PROTAC degraders.

### 4.2 The utilizations in PROTACs

#### 4.2.1 AR

Nutlin‐3 (Figure 4; MDM2‐1) is a potent MDM2 inhibitor that bind to the p53‐binding pocket of MDM2.^348^, ^349^ In 2008, Nutlin‐3 were also used for the design of the first all‐small‐molecule type PROTAC‐12 to recruit endogenous MDM2 for targeting AR by Crews group (Table 1).^5^

#### 4.2.2 BRD4

In 2019, The Crews group developed an MDM2/nutlin‐based BRD4 degrader A1874 with potent degradation on BRD4 protein and inhibition on the growth of cancer cells harboring a wild type p53 (Table 1 and Figure 4; MDM2‐2).^350^

#### 4.2.3 PARP1

The nuclear protein PARP1 has a clear role in DNA damage response and repair and is an effective therapeutic target for human cancers and other human diseases.^351^, ^352^ Although some small‐molecule PARP1 inhibitors, such as Olaparib and Rucarparib, have been approved by the US FDA to treat ovarian and breast cancer with BRCA mutations, there are still significant challenges such as developed drug resistance limiting their efficacy. TPD is a promising strategy to solve this problem.

In 2018, The Rao group developed the first‐in‐class PARP1‐targeting PROTAC‐13 (Table 1) by connecting the PARP1 inhibitor niraparib to the MDM2 ligand nutlin‐3 (Figure 4; MDM2‐3).^353^ Specifically, Compound PROTAC‐13 selectively and significantly induced PARP1 degradation and cell apoptosis in MDA‐MB‐231 cells with a fivefold increased potency in antiproliferative activity, as compared with the PARP1 inhibitors niraparib, olaparib, or veliparib alone, while showing no cytotoxicity in normal cells.^353^

#### 4.2.4 MDM2

In 2021, Sheng group developed the first‐in‐class homo‐PROTAC‐14, targeting MDM2 by inducing its self‐degradation (Table 1 and Figure 4; MDM2‐1/MDM2‐4). The compound PROTAC‐14 efficiently induced the dimerization of MDM2 in A549 NSCLC cells, thereby inducing the self‐degradation of MDM2 through the proteasome system, harboring a wild‐type p53.^278^

Given an extensive list of small molecule MDM2 inhibitors, the development of MDM2‐based PROTAC degraders should have more opportunity for clinical application.

## 5 IAP LIGANDS AND THEIR UTILIZATIONS IN PROTACS

### 5.1 Common IAP ligands

**FIGURE 5:**
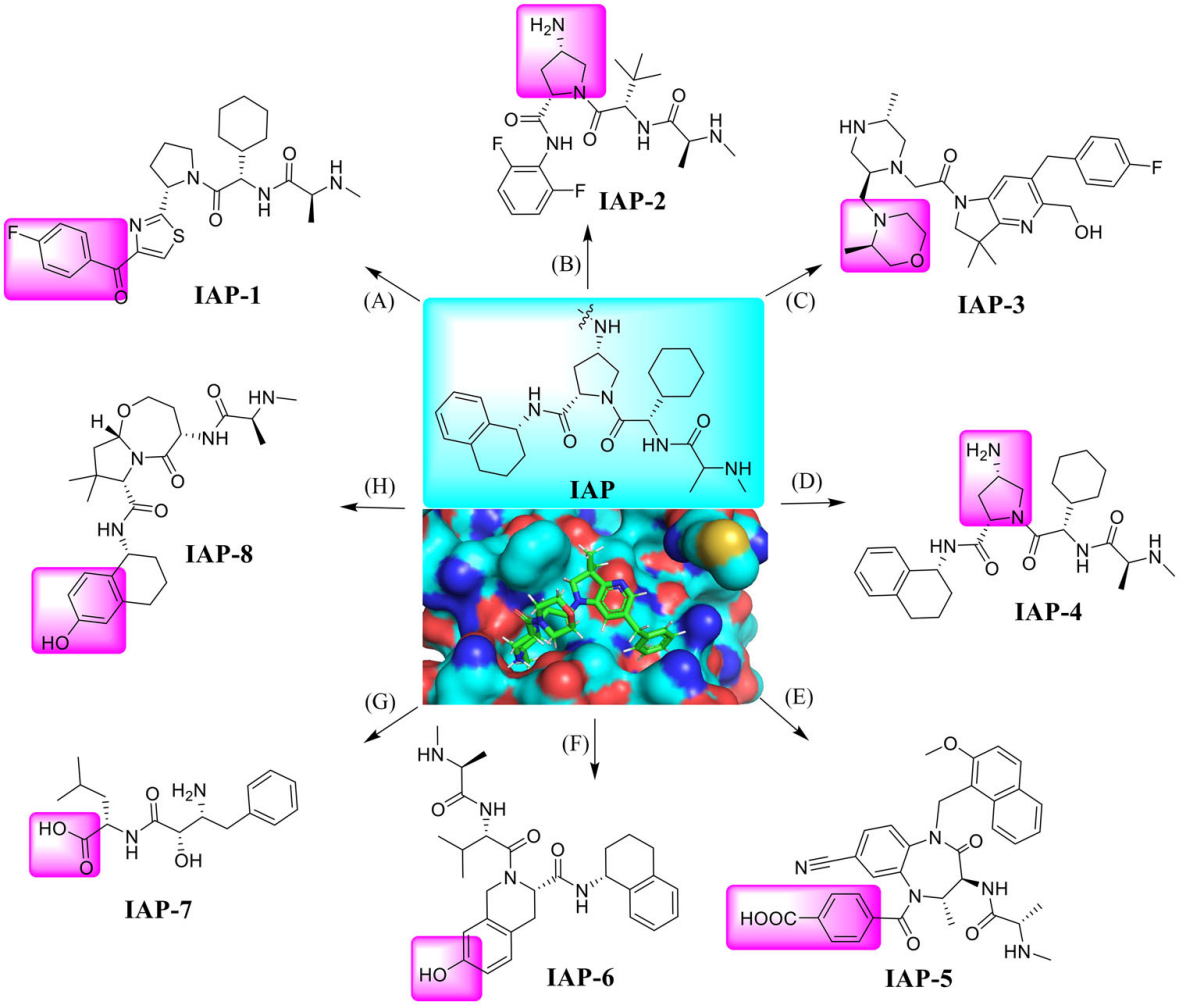
A variety of small molecules that serve as the ligands for IAP E3 ligase. (A–H) Various small molecules (IAP‐1–8) acting as IAP E3 ligase ligands (PDB ID: 5M6H).

cIAP1, cIAP2, and X‐chromosome‐linked IAP (XIAP) belong to the family of antiapoptotic proteins that play a critical role in the control of apoptotic machinery.^354^, ^355^, ^356^, ^357^, ^358^, ^359^, ^360^, ^361^ The widespread use of IAPs as an E3 ligase in TPD has drawn much attention from scientists in both academia and industry.^362^, ^363^, ^364^, ^365^, ^366^, ^367^, ^368^ Correspondingly, many IAPs inhibitors were discovered and applied in the design of PROTAC degraders.^284^ Specifically, the pink moiety is exposed to the solvent as a potentially ideal linking position in the design of PROTAC degraders, based on the crystal structure of IAPs inhibitor with IAPs protein (Figure 5; PDB ID: 5M6H).^369^, ^370^

### 5.2 The utilizations in PROTACs

#### 5.2.1 AR

IAP‐targeting heterobifunctional degraders were also named as specific and nongenetic IAP‐dependent protein erasers (SNIPERs). In 2010, Hashimoto described the first concept of SNIPER.^363^ In 2018, the Naito group reported AR degrader PROTAC‐15, which were named SNIPERs (Table 1). Specifically, compound PROTAC‐15 was designed using an AR antagonist and a ligand IAP‐1 (Figure 5) for the cellular inhibitor of apoptosis protein 1 (cIAP1) as the E3 ligase.^371^

#### 5.2.2 RIPK2

Receptor interaction serine/threonine kinase 2 (RIPK2) is a single‐channel transmembrane protein receptor with intracellular serine/threonine protein kinase activity.^372^ In 2021, the Harling group reported a series of PROTAC RIPK2 degraders which recruit members of the inhibitor IAP‐2 (Figure 5) of apoptosis family of E3 ligases.^373^ Specifically, the authors identified a series of IAP binding compounds and have successfully applied to the new and highly effective RIPK2 PROTACs PROTAC‐16 and PROTAC‐17 (Table 1).

#### 5.2.3 BTK

Bruton's tyrosine kinase (BTK), nonreceptor cytoplasmic tyrosine kinase, is a key regulator in the B‐cell receptor (BCR) signaling pathway.^374^, ^375^ BTK plays a key role in B‐cell lymphomas and is a validated target for mantle cell lymphoma (MCL). Although BTK inhibitors are clinically effective in cancer therapy, drug resistance remains a major challenge.^374^, ^376^ For example, ibrutinib, a covalent inhibitor approved by the US FDA in 2013, was used to treat MCL and ABC‐DLBCL in patients who have developed resistance due to a C481S missense BTK mutation.^377^

Currently, a variety of noncovalent degraders have been developed that can effectively degrade wild‐type and Ibrutinib‐resistant C481S BTKS, which may be an effective solution to the problem of serious resistance to BTK inhibitors. IAP‐4 (Figure 5) was used in the discovery of PROTAC BTK degraders by the Harling group in 2019. Reversible compound PROTAC‐18 (Table 1) caused BTK degradation effectively and reduced cIAP1 levels at concentrations of >30 nM.^378^ In 2020, Calabrese group designed and synthesized a potent BTK degrader BCPyr (Table 1) by linking amino‐pyrazole derivatives to IAP ligand IAP‐6 (Figure 5) with a DC_50_ of 800 nM.^379^

#### 5.2.4 ER

IAP‐5 (Figure 5), another type of IAP ligand, was used in efforts by Genentech Inc. to develop a series of potent degraders of the ERα, as exemplified by PROTAC‐19 (Table 1). In this report, the authors described a novel application of antibody‐drug coupling technologies that effectively delivered chimeric ERα degrader molecules to targeted MCF7 cells.^380^

#### 5.2.5 BCR–ABL

In 2016, compound SNIPER‐2 (Table 1) was reported as a degrader targeting BCR–ABL by linking a BCL‐ABL inhibitor imatinib derivative to IAP‐7 (Figure 5) that binds to cIAP147.^381^

#### 5.2.6 BCL‐X_L_

BCL‐X_L_ is an antiapoptotic BCL2 (B‐cell lymphoma) family member and its overexpression is a key marker of partial escape from apoptosis in cancer.^382^ Studies have shown that BCL‐X_L_ is a very effective cancer target. Inhibition of BCL‐X_L_ protein has been widely recognized as a promising strategy for cancer therapy, and several representative anticancer drug candidates in the BCL‐X_L_ inhibitor class have been produced.^383^ Although these inhibitors are effective in the treatment of certain hematological malignancies such as CLL and AML, BCL‐X_L_ inhibitors still present significant challenges, such as developed resistance and dose limitations that limit their clinical efficacy.^384^ Thus, there is an urgent need to develop a new approach to the development of BCL‐X_L_ targeted drugs, and PROTAC technology comes to the stage. In 2020, the Zheng group developed a series of PROTACs by recruiting IAP E3 ligase (Figure 5; IAP‐8) for BCL‐X_L_ degradation, as exemplified by compound PROTAC‐20 (Table 1).^385^ BCL‐X_L_ can be degraded by compound PROTAC‐20 powerfully in multiple cancer cells.

Future development of IAP‐based PROTAC degraders targeting other disease‐associated proteins is expected.

## 6 KEAP1 LIGANDS AND THEIR UTILIZATIONS IN PROTACS

### 6.1 Common KEAP1 ligands

**FIGURE 6:**
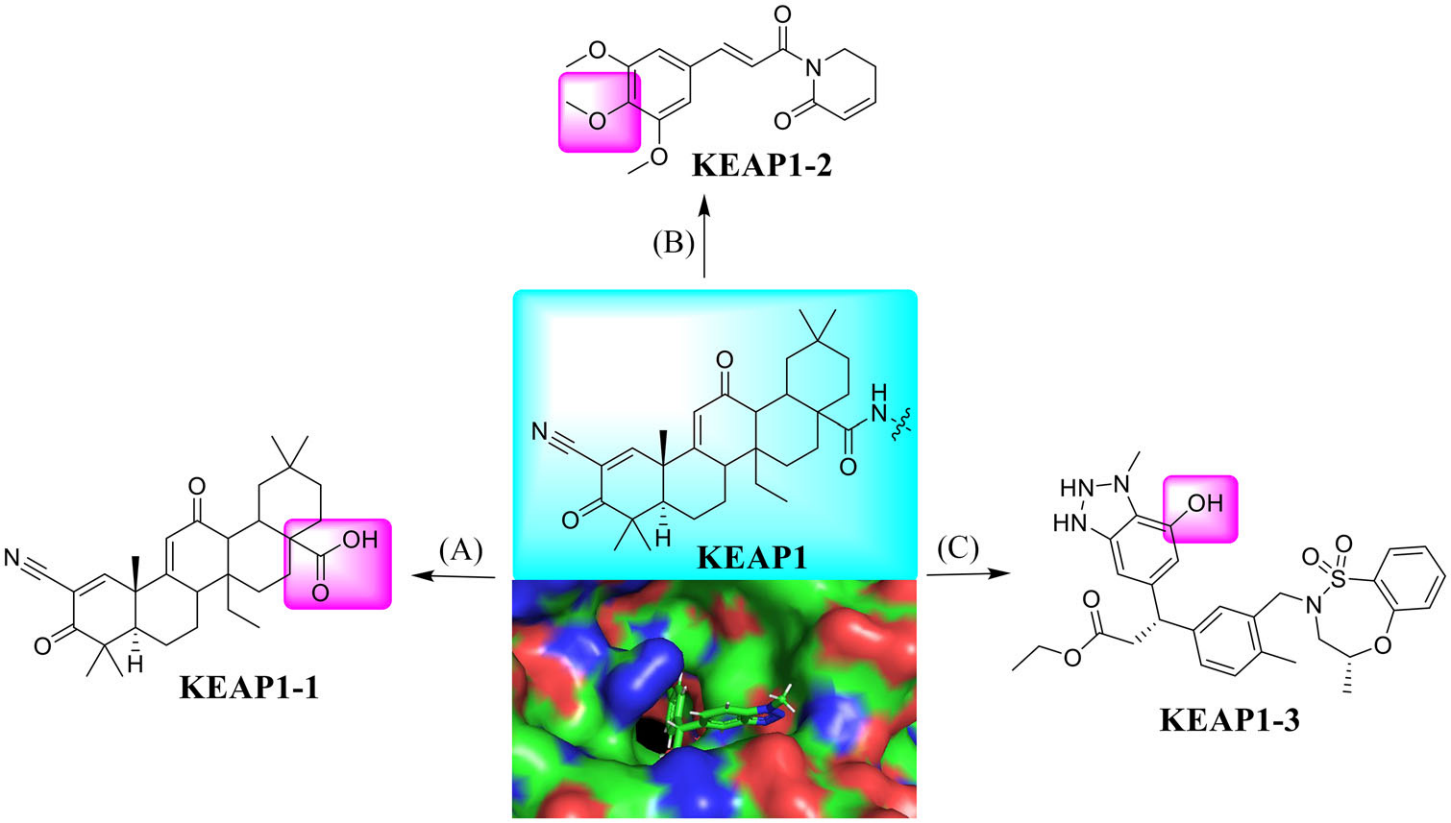
A variety of small molecules that serve as the ligands for KEAP1 E3 ligase. (A–C) Various small molecules (KEAP1‐1–3) acting as KEAP1 E3 ligase ligands (PDB ID: 6QMD).

The KEAP1 is a substrate‐recognizing subunit of cullin RING ligase (CRL), responsible for ubiquitylation and subsequent degradation of antioxidant transcription factor nuclear factor erythroid 2‐related factor 2 (Nrf2).^26^, ^386^, ^387^, ^388^, ^389^ Structurally, KEAP1 mainly consists of an N‐terminal bric‐a‐brac of tramtrack, a broad complex (BTB) domain for cullin 3 binding, and a C‐terminal Kelch domain for substrate recruitment.^390^, ^391^, ^392^, ^393^ Recently, a number of small molecules have been shown to modulate the KEAP1/NRF2 axis (Figure 6).^284^, ^394^, ^395^, ^396^, ^397^, ^398^ The BTB domain inhibitors, including natural products KEAP1‐1 (a semi‐synthetic oleanolic acid derivative bardoxolone) and KEAP1‐2 (piperlongumine), were discovered as a promising NRF2 modulators utilized in TPD applications by covalently interacting with cysteine residues of KEAP1.^399^ On the other hand, a Kelch domain reversible inhibitor, designated as KEAP1 KEAP1‐3, was also discovered as an E3 ligand.^284^ The pink portion with exposure to the solvent zone is proposed as a potentially ideal linking position in the design of KEAP1‐based PROTACs (Figure 6; PDB ID: 6QMD).^400^

### 6.2 The utilizations in PROTACs

#### 6.2.1 BRD4

In 2018, Lu group developed the first‐in‐class KEAP1‐dependent peptide PROTAC.^401^ However, the peptide binding component of KEAP1 greatly limits its potential in TPD applications. In 2020, the Daniel group discovered a BRD4‐targeting PROTAC based on the BTB ligand KEAP1‐1, as exemplified by compound CDDO‐JQ1 (Table 1 and Figure 6; KEAP1‐1).^399^ In 2021, the Jin group reported that the KEAP1‐1 E3 ligase ligand (Figure 6) can be designed for TPD of BRD2/3/4 proteins, as exemplified by compound MS‐83 (Table 1).^402^

#### 6.2.2 CDK

Recently, the Lv group developed a type of PROTAC CDK9 degrader PROTAC‐21 on preprint bioRxiv via hijacking piperlongumine KEAP1‐2 (Figure 6) as a new type of E3 ligand to induce TPD (Table 1).^403^ The optimized compound PROTAC‐21 could effectively induce the degradation of CDK9 with a good DC_50_ value of 9 nM in MOLT4 cell line. Currently, the reported KEAP1‐based PROTAC degraders are still very limited and more studies are needed to evaluate its potential in clinical application.

## 7 OTHER TYPES OF E3 LIGASE LIGANDS

### 7.1 DCAF ligands and their utilizations in PROTACs

#### 7.1.1 Common DCAF ligands

**FIGURE 7:**
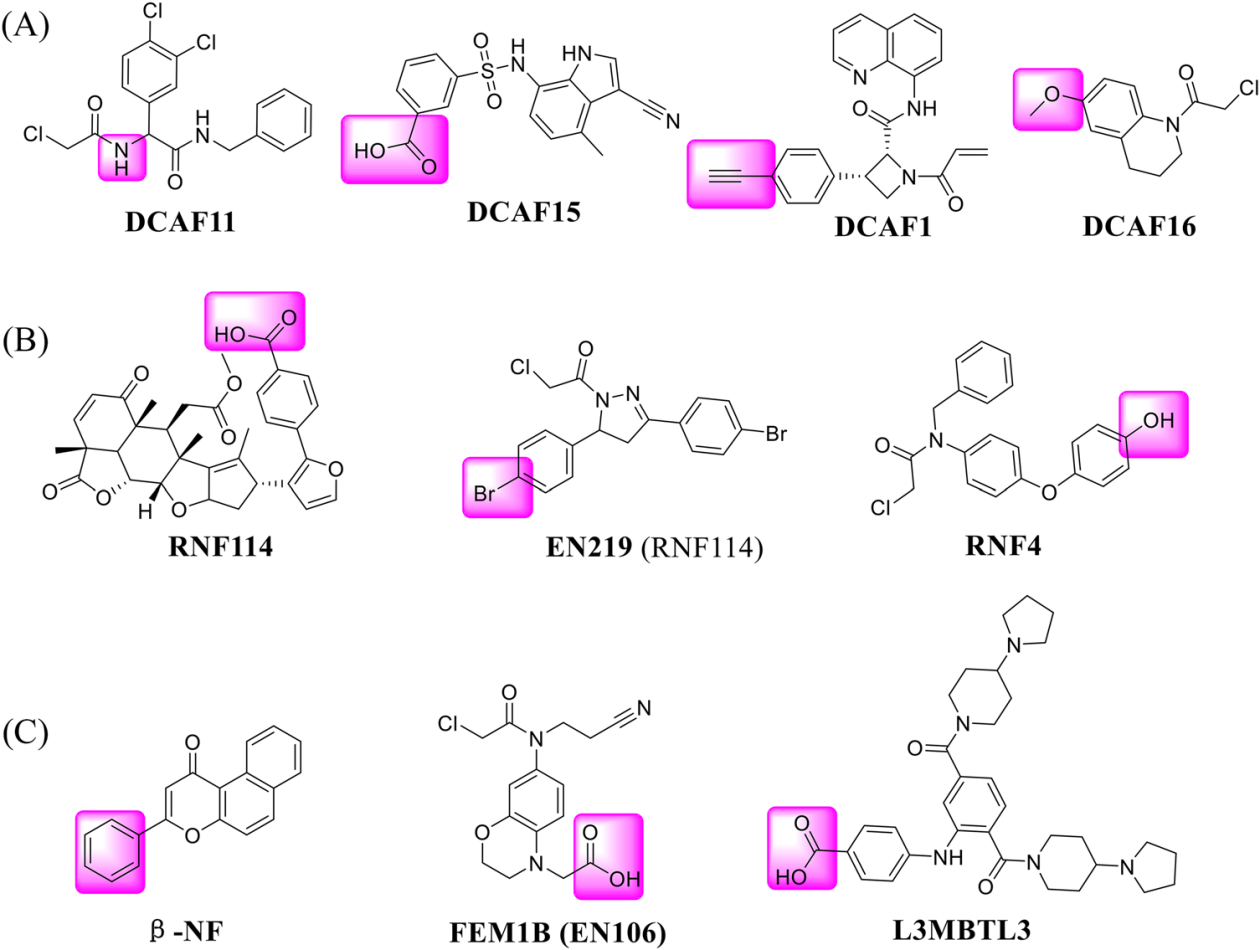
Other types of the ligands for few E3 ligases. (A) Novel DCAF CRL4 E3 ligase ligands used in PROTACs. (B) Novel RNF SUMO E3 ligase ligands used in PROTACs. (C) Novel FEM1B CRL2 E3 ligand, L3MBTL3 Cul4^DCAF5^ E3 ligand, and β‐NF E3 ligand used in PROTACs.

DCAF is located in the cell nucleus and represents a substrate recognizing subunit of the CRL4 E3 ligases, including DCAF11, DCAF15, DCAF1, and DCAF16.^404^, ^405^, ^406^ Although the lack of structural information makes it difficult for further optimization of DCAF derivatives, the corresponding ligands have been successfully used in PROTAC (Figure 7A).

#### 7.1.2 The utilizations in PROTACs

*FKBP12*: FK506 binding protein 12 kDa (FKBP12) is a evolutionarily conserved protein with peptidyl prolinase (PPIase) activity existing in eukaryotes.^407^ FKBP12 is an effective target for drug development, including immunosuppressive drugs tacrolimus (FK506) and Sirolimus (rapamycin).^408^ In 2019, the Cravatt group first reported a new E3 ligase ligand identified as DCAF16 (Figure 7A) and applied it in the discovery of PROTAC FKBP12 degrader KB02‐SLF (Table 1).^406^ The same group reported a functional screening strategy performed in a focused library for candidate electrophilic PROTACs, and discovered bifunctional compounds that degrade proteins in human cells via covalently engaging DCAF11 ligand (Figure 7A), as exemplified by compound 21‐SLF in 2021 (Table 1).^409^

*BRD4*: Most recently, Cravatt group continued to expand the scope of DCAF E3 ligands and discovered a new type of E3 ligand reacting with a cysteine (C1113) in the E3 ligase substrate receptor DCAF1 (Figure 7A), leading to identification of the new DCAF1 E3 ligand for targeted BRD4 degradation with compound YT117R (Table 1).^410^ On the other hand, in 2020, Chen and Li group developed a new type of DCAF15‐hijacking BRD4 PROTAC DP‐1, based on DCAF15 ligand sulfonamide E7820 that was linked to a pan‐BET inhibitor JQ‐1 (Table 1 and Figure 7A).^411^

However, the studies engaging DACF family E3 ligands are very limited at the present time.

### 7.2 RNF ligands and their utilizations in PROTACs

#### 7.2.1 Common RNF ligands

RNF114 contains a C‐terminal ubiquitin interaction motif and an N‐terminal RING domain, both of which are associated with its E3 ubiquitin ligase activity,^412^, ^413^ whereas RNF4 is a small ubiquitin‐like modifier (SUMO)‐targeted ubiquitin ligase.^414^, ^415^, ^416^ Nimbolide, a derivative of natural product, was identified as an effective E3 ligand targeting RNF114 protein.^413^ Since then, EN219, a fully synthetic RNF114 binder, was also employed as the RNF114 recruiter in the design of PROTAC degraders.^417^, ^418^, ^419^, ^420^, ^421^, ^422^ Recently, the covalent binder, compound RNF4 was discovered as a new RNF4 E3 ligase recruiter (Figure 7B).^423^

#### 7.2.2 The utilizations in PROTACs

*BRD*: The studies using RNF114 or RNF4 as the E3 ligases for PROTAC degraders were exclusively from the Nomura group. In 2019, the group identified a terpenoid natural product Nimbolide as a novel RNF114 E3 ligase ligand (Figure 7B) to recruit RNF114 for targeted degradation of BRD4 protein with compound XH‐2 (Table 1).^413^ In 2021, again the same group identified a fully synthetic covalent ligand EN219 (Figure 7B) that targets RNF114 E3 ligase to effectively degrade BRD4 protein with compound ML 2−14 (Table 1).^417^ Furthermore, the Nomura group developed cysteine‐reactive small‐molecules that react with the E3 ubiquitin ligase RNF4 by utilizing activity‐based protein profiling (ABPP)‐based covalent ligand screening approaches, and demonstrated that the BRD4 degrader CCW 28‐3 could effectively induce the degradation of BRD4 protein in a proteasome‐ and RNF4‐dependent manner (Table 1 and Figure 7B).^423^ This series of exploratory findings have expanded the range of E3 ligase recruiters for PROTAC‐based TPD. In 2022, Nomura group reported a cysteine‐reactive covalent ligand, EN106, that recruited FEM1B, an CRL2 substrate‐recognizing subunit responsive to the cellular reductive stress (Figure 7C),^424^ and showed that a PROTAC NJH‐1‐106 (Table 1) connecting EN106 with the pan‐BET inhibitor JQ1 or the kinase inhibitor dasatinib triggered the degradation of BRD4 and BCR–ABL, respectively.^425^ Very recently, Crews group reported a novel approach to target protein degradation by hijacking a methyl‐lysine reader protein L3MBTL3, which binds to the Cul4^DCAF5^ E3 ligase complex (Figure 7C).^426^ Here, L3MBTL3 E3 ligand was employed as the L3MBTL3 E3 ligase recruiter in the design, synthesis and biological evaluation of KL‐7 and KL‐4 of BRD2 and FKBP12 proteins, respectively (Table 1). The authors proposed this approach as a general way to extend the E3 ligase toolbox and to explore the full potential of TPD by utilizing E3 ligase complexes associated with other reader proteins in PROTAC designs.^426^ This will make already large‐sized PROTAC degrader even bigger.

*BCR–ABL*: In 2020, the Nomura group also applied RNF114 E3 ligase ligand (Figure 7C) to discover PROTAC BCR–ABL degrader BT‐1, and demonstrated that nimbolide‐recruited BCR–​ABL bifunctional compound selectively degraded BCR–​ABL over c‐​ABL in leukemia cancer cells (Table 1).^427^ Compound BT‐1 induced the degradation of BCR–ABL with a DC_50_ value of 1 μM in K562 cell line.

*CRABPs*: Cellular retinoic acid binding proteins (CRABPs) are a class of high‐affinity retinoid‐binding proteins mainly found in the cytoplasm.^428^ Two members of CRABPs, CRABP‐I, and CRABP‐II, are found in all vertebrates and conserved between species.^429^ Research has linked CRABP‐I/II to Alzheimer's disease and many cancers. CRABPs may be promising targets for these diseases.^430^ However, direct inhibition of CRABPs' function by small molecule inhibitors remains challenging. In 2019, the Naito group reported an AhR ligand (β‐NF) (Figure 7C) recruiting the AhR E3 ligase complex to develop a novel PROTAC β‐NF‐ATRA (Table 1). β‐NF‐ATRA, a PROTAC that recruits CRABPs, induces CRABPI and CRABPII degradation in an AhR‐dependent manner via the UPS pathway, which may lead to potential synergistic antitumor activity.^431^

## 8 CONCLUSION AND PROSPECTS

As a revolutionary technology in the drug discovery and development, TPD, mainly by the PROTAC and molecular glue, has been utilized to successfully degrade a variety of pathogenic proteins, including oncogenic proteins, viral and bacterial proteins, and has been widely used for potential targeted therapy of a variety of human diseases such as cancers, infectious diseases, neurodegenerative diseases, autoimmune diseases, among others. At present, ARV‐471, a PROTAC degrader of ER for the treatment of breast cancer, is at the phase III clinical trial, along with few dozens of degraders, having entered or about to enter the early phase of clinical trials. The field is blooming with many PROTAC degraders competing for the clinical market.

In the past two decades, although scientists have developed few E3 ligase ligands and successfully applied them to the discovery and development of PROTAC degraders, the highly utilized E3 ligands were mainly limited to VHL and CRBN E3s, a family of multicomponent cullin‐RING ligases. In this review, we summarized approximate 10 reported E3 ligases and a variety of paired small molecule ligands currently used in PROTAC degraders and their applications for degradation of different oncogenic targets. A major challenge for further extension of PROTAC technology in drug discovery is to discover and develop more potent and selective small molecule ligands to couple with a variety of E3 ubiquitin ligases with a family of more than 600 members in the human genome. To this end, two feasible approaches should be fully employed to identify and characterize more potent and selective small molecule ligands for additional E3 ubiquitin ligases: (1) the AI‐based structure prediction of E3 ligases, coupled with computer‐based docking/virtue‐screening; (2) the DNA‐encoded library screening of novel E3 ligase ligands. The breakthrough in discovery of more E3 ligands will certainly expand current scope in the development of effective PROTAC degraders for eventual treatment of various human diseases.

## AUTHOR CONTRIBUTION

X. H. drafted and Y. S. revised/finalized the manuscript. All authors have read and approved the final manuscript.

## CONFLICT OF INTEREST STATEMENT

The authors declare that they have no conflicts of interest in this work.

## ETHICS STATEMENT

No ethical approval is required.

## Data Availability

All data are freely available from the corresponding author upon request.
